# Adverse Events Associated With Treatment of *Tripterygium wilfordii* Hook F: A Quantitative Evidence Synthesis

**DOI:** 10.3389/fphar.2019.01250

**Published:** 2019-11-06

**Authors:** Yi Ru, Ying Luo, Yaqiong Zhou, Le Kuai, Xiaoying Sun, Meng Xing, Liu Liu, Yi Lu, Seokgyeong Hong, Xi Chen, Jiankun Song, Yue Luo, Xiaoya Fei, Bin Li, Xin Li

**Affiliations:** ^1^Department of Dermatology, Yueyang Hospital of Integrated Traditional Chinese and Western Medicine, Shanghai University of Traditional Chinese Medicine, Shanghai, China; ^2^Institute of Dermatology, Shanghai Academy of Traditional Chinese Medicine, Shanghai, China

**Keywords:** *Tripterygium wilfordii* tgpolyglycoside, *Tripterygium wilfordii* Hook F, adverse events, meta-analysis, systematic review

## Abstract

**Background:** *Tripterygium wilfordii* Hook F can cause adverse effects (AEs) in clinical application and may be harmful to human health. This study aim to summarize the AEs caused by *T. wilfordii* tgpolyglycoside (TWP), the most common preparation of *T. wilfordii* Hook F for clinical use.

**Methods:** The Cochrane Library, EMBASE, PubMed, and Web of Science were searched to identify potential articles on this topic. All single-arm trials, controlled clinical trials, and randomized controlled trials were selected and summarized. Meta-regression was used to determine the sources of heterogeneity, and subgroups were used to identify factors leading to AEs.

**Results:** Forty-six studies, comprising 25 randomized controlled trials, 13 controlled clinical trials, and 8 single-arm trials, were included in this meta-analysis, representing 2437 enrolled TWP-treated participants. Combined intervention, drug dosage, medication treatment, pharmaceutical manufacturers, and specific organ toxicity were identified as potential factors leading to TWP-induced AEs in this meta-analysis. In patients treated with TWP, the global incidence of AEs was 30.75% (95% confidence interval [21.18–40.33], *I* ^2^ = 97%), and that of severe grade AEs was 4.68% (95% confidence interval [0.00–12.72], *I* ^2^ = 53%). Organ-specific analyses indicated that TWP treatment elicited intestinal toxicity, reproductive toxicity, hepatotoxicity, nephrotoxicity, hematotoxicity, cutaneous toxicity, and other damages. The AEs analyzed in the subgroups of combined intervention, drug dosage, medication treatment, and pharmaceutical manufacturers were considered as primary outcomes, and organic-specific AEs were considered as secondary outcomes.

**Conclusions:** The occurrence of TWP-induced AEs was systemic, organ-specific, and related to medication course, combined intervention, and drug dosage.

## Introduction

*Tripterygium wilfordii* Hook F (TwHF), a vine plant, is used as a Chinese herbal medicine (CHM). Currently, the root of TwHF is the main officinal part used in medicinal applications, although its stem and leaf also have the same chemical composition and function as those of the root. TwHF has anti-inflammatory, anti-fertility, anti-tumor, antibacterial, (Reno et al., 2015) and other biological activities. It has determinate and curative effects on immune disorders and is an efficient substitute drug for autoimmune diseases (Liu et al., 2019). Owing to the adverse effects (AEs) of TwHF in clinical preparations, methods for reducing the toxicity and increasing the efficacy of TwHF are being extensively investigated. In order to unify TwHF preparations in the statistics, we analyzed studies that included only the use of *T. wilfordii* tgpolyglycoside (TWP) treatment. TWP is the most widely used TwHF preparation in clinical application for kidney disease, rheumatism, Crohn’s disease, skin diseases, thyroid problems, and Sjogren’s syndrome (Luo et al., 2019). Several active ingredients such as triptolide, triptonide, celastrol, wilforlide A, and wilforine are contained in TWP, which extracted from TwHF with elimination of the main toxic components (Tian et al., 2012). Despite the sophistication of the drug purification process, related AEs still occur intermittently.

Different types of complex and challenging AEs have evolved owing to the wide clinical application of TwHF. Previous studies have shown that the AEs caused by TWP mainly include leukopenia, gastrointestinal reactions, menstrual disorders, and liver dysfunction (Zhang et al., 2016). Incidences of leukopenia and liver dysfunction have significantly increased in patients treated for immune system disease, and independent incidences of AEs caused by TwHF have been associated with various organ toxicities, such as intestinal toxicity, reproductive toxicity, hepatotoxicity, nephrotoxicity, hematotoxicity, and cutaneous toxicity (Liu et al., 2018; Zhang et al., 2018). This indicates that the therapeutic dose of TwHF is close to its toxic dose, resulting in unavoidable occurrence of AEs. However, its safety can be controlled within acceptable limits (Wang et al., 2018; Zhou et al., 2018). Hence, the China State Food and Drug Administration issued a drug warning on TwHF preparations in April 2012 to restrict its clinical application (The State Food and Drug Administration, 2012).

Although some patients experience AEs due to TwHF, reasonable treatment and management strategies can help improve their tolerance to the plant. The dosage and treatment courses of TwHF should be strictly controlled, and patients treated with TwHF should be closely observed for AEs. TwHF-induced AEs attracted public attention for the first time in 2013 (Chen et al., 2013). However, researchers have focused on the incidence of AEs in a single systemic disease (primary nephrotic syndrome), although TwHF is a multi-systemic drug used for treating multi-systemic diseases. Therefore, high-quality meta-analyses of the AEs associated with TwHF are needed.

TWP, the most common preparation of TwHF in clinical practice, was the object of analysis of this study. We collected and analyzed articles on AEs caused by TWP and investigated the main AEs and risk factors associated with TwHF to provide references for future clinical safety.

## Methods

This systematic review was performed following the Cochrane Handbook for Systematic Reviews of Interventions, (Higgins and Green, 2018) presented under the Preferred Reporting Items for Systematic Reviews and Meta-analyses (PRISMA) guidelines shown in **Supplementary Table 3**.

### Selection Criteria

In this analysis, we included studies of randomized controlled trials (RCTs), controlled clinical trials (CCTs), and single-arm trials reporting AEs after the intervention of TWP, irrespective of sex, age, and ethnicity.

The exclusion criteria were as follows: (i) studies on non-TWP extract or compound; (ii) studies not reporting AEs; (iii) articles of meeting abstracts, cell or animal studies, reviews, systematic reviews, and meta-analysis; and (iv) articles with full text not available.

### Outcomes

The primary outcomes considered in this study were the incidence of AEs and their grades, which were recorded according to the Common Terminology Criteria for Adverse Events 5.0 (CTCAE 5.0) of the National Cancer Institute. AE grades ≥3 were considered severe. AE incidence was evaluated in terms of drug dosage, treatment duration, combined interventions, and pharmaceutical manufacturers as the primary outcome. Organ-specific AEs were measured as the secondary outcomes.

### Selection of Studies and Data Extraction

The Cochrane Library, Excerpta Medica Database (EMBASE), PubMed, and Web of Science (WOS) were searched in this study. Studies dating from the earliest citation in the databases till November 2018 were included irrespective of language. The search terms used were “safety,” “side effects,” and “adverse events,” combined with “*Tripterygium wilfordii* Hook F.,” “*Tripterygium wilfordii* tgpolyglycoside,” “Lei Gong Teng,” and “clinical.”

Three investigators independently screened the studies according to the inclusion criteria using self-designed data-extraction templates, and extracted information that included the first author, study characteristics (article type, year, and design), drug characteristics (medication dosage, pharmaceutical manufacturers, combined interventions, administration route, and drug duration), participant characteristics (region, sex, mean age, sample size, and diagnosis), and AE characteristics (incidence rate, occurrence time, specific AE manifestations, and solutions), and then measured the outcomes. Two authors assessed the risk of bias, three authors performed data analysis and interpretation, four authors performed statistical analyses, three authors drafted the manuscript, and all authors read and approved the final manuscript.

### Quality Assessments

The Cochrane Handbook (Higgins and Green, 2018) was used to evaluate the methodological quality of the included RCTs regarding the following characteristics: random sequence generation, allocation concealment, blinding of participants and personnel, blinding of the outcome assessment, incomplete outcome data, selective reporting, and other biases. The terms “low,” “unclear,” and “high” referred to low, uncertain, and high risks of bias, respectively. The quality of non-RCTs was evaluated using the Newcastle–Ottawa Scale (NOS) (Stang, 2010) The results were cross-checked by two investigators, and disagreements were settled *via* discussion.

### Statistical Analysis

The incidence of AEs was estimated for the studies included in this meta-analysis. We pooled the incidence of AEs in TWP treatment. Heterogeneity between studies was assessed using the Q test and *I* ^2^ statistics. When the heterogeneity was small, that is when *I* ^2^ < 50%, the fixed-effect model was used for analysis, and when the heterogeneity was large, that is when *I* ^2^ < 50% and *P* > 0.1, the random-effect model was used for analysis. The overall heterogeneity of the results in this meta-analysis was large; hence, the random-effect model was used to calculate the average statistics of the weighted combination of multiple research statistics. Potential publication bias was examined using funnel plots. Incidence and meta-regression analyses were performed using R software [version 3.8.6 (2018–3–15); Nordic Cochrane Centre, Copenhagen, Denmark] with the package Meta and Metafor function.

## Results

### Study Selection

**Figure 1:**
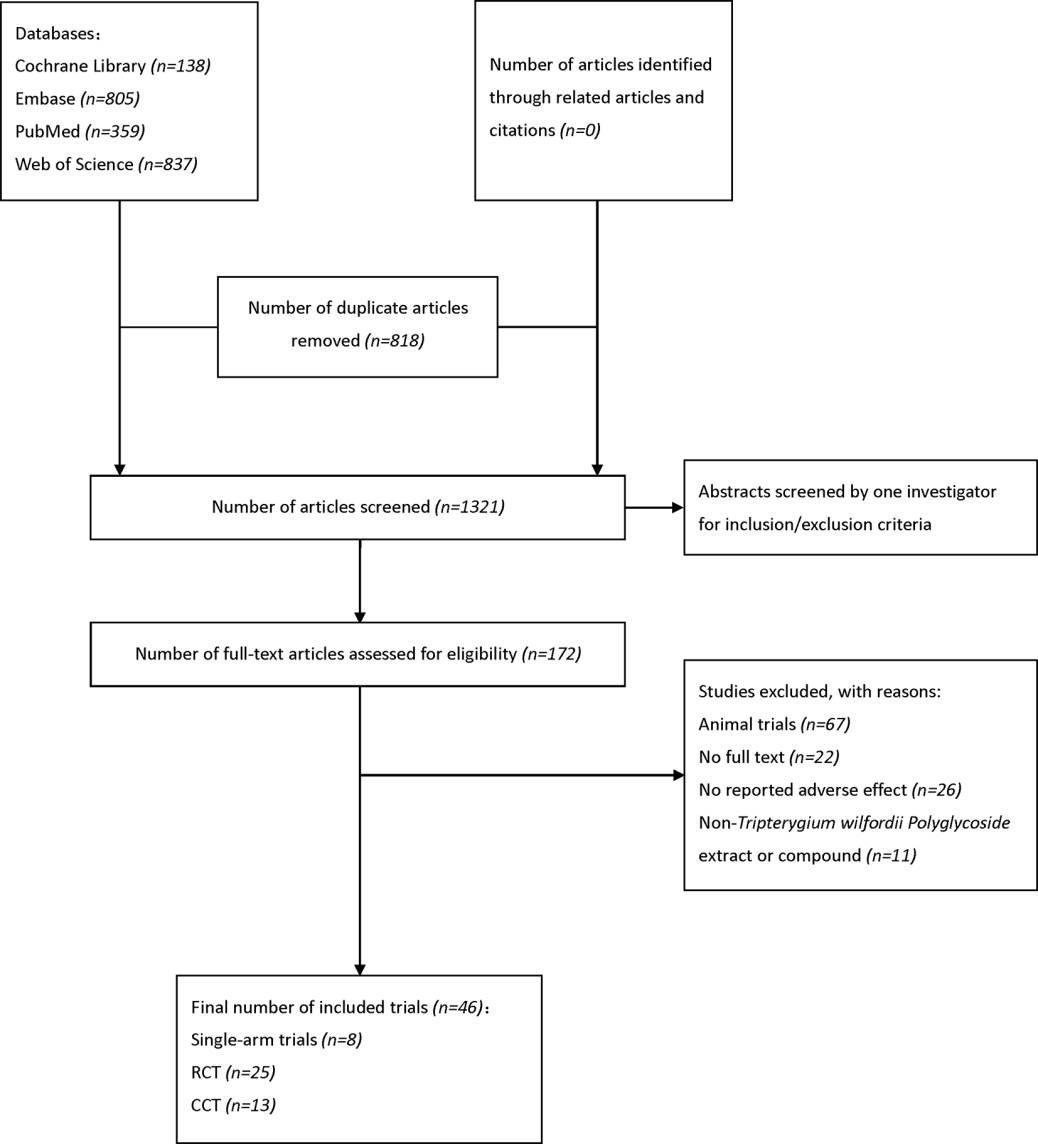
Flow diagram of literature search and selection. EMBASE, Excerpta Medica Database; RCT, randomized controlled trial; CCT, controlled clinical trial.

Our search strategy identified 2138 potential studies from electronic databases, and the detailed steps are shown in **Figure 1**. In total, 818 articles were excluded because of duplication and 1321 studies were reserved temporarily. The remaining articles were screened for titles and abstracts, and 172 studies were saved based on our inclusion and exclusion criteria. Furthermore, 126 studies were excluded as they did not meet our expectations upon perusing the entire text. Finally, we included 46 studies (Ao et al., 1994; Jiang, 1994; Zhang et al., 1994; Tao et al., 1995; Ji et al., 1998; Zhou et al., 1999) in the meta-analysis.

### Study Characteristics

**Figure 2:**
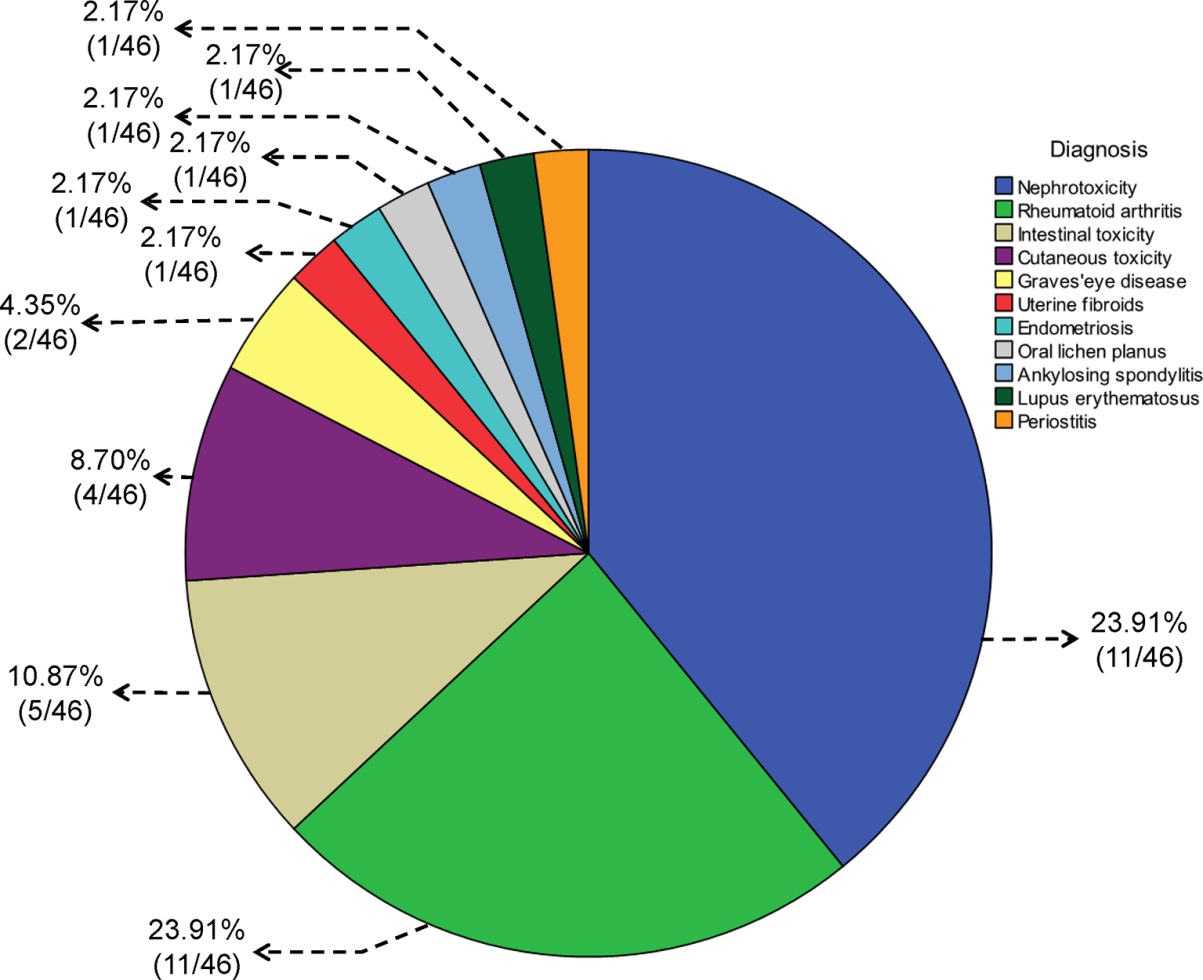
Pie chart for the diagnosis of diseases treated with *Tripterygium wilfordii* tgpolyglycoside.

**Figure 3:**
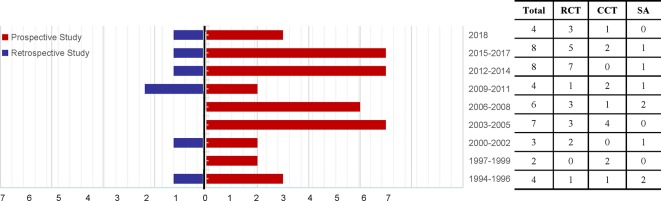
Time trend of the 46 articles relevant to *Tripterygium wilfordii* tgpolyglycoside-induced adverse events (prospective studies vs. retrospective studies), search performed until November 2018. RCT, randomized controlled trial; CCT, controlled clinical trial; SA, single-arm trial.

Specific details of the clinical trials are presented in **Table 1** and **Figure 2**. Overall, 2437 enrolled TWP-treated participants were included in the meta-analysis. There were 25 RCTs, 13 CCTs, and 8 single-arm trials. Seven of these were retrospective studies and 39 were prospective studies (**Figure 3**). Among the 46 studies, at least one study used TWP as a treatment approach, which was defined as an experimental group or a control group. The sample size of the 46 trials ranged from 1 to 210. Twenty-one studies (with 1075 patients) used TWP as the primary and only treatment approach. Twenty-nine studies (with 1441 patients) used TWP as the active group, and 15 studies (with 658 patients) were used as the control group.

**Table 1:** Characteristics of the included trials.

Studies	Article type	Age/Mean [SD]	Region	Treatment centre	Sample size	Diagnosis	Total incidence of AEs (number)	Source	Species, concentration (Triptolide; Tripterifordin; Celastrol, μg/g) (Luo et al., 2016)	Quality control reported (Y/N)	Chemical analysis reported (Y/N)
Ao et al. (1994)	CCT	41.83 [1.02]	China Beijing	Department of Urology, Chinese PLA General Hospital	87	Kidney transplantation	NR	MT	9.28; 105.06; 402.88	Y-WS3-98	Y-HPLC (Wu et al., 2015)
Jiang (1994)	Single-arm	NR	China Jiangsu	NR	13	Nephrotic syndrome	92.31% (Stang, 2010)	MT	9.28; 105.06; 402.88	Y-WS3-98	Y-HPLC
Zhang et al. (1994)	RCT	29.5 [13.2]	China Shanghai	NR	30	Islet transplantation	10% (Tian et al., 2012)	FH	9.78; 92.46; 398.45	Y-WS3-98	Y-HPLC
Tao et al. (1995)	Single-arm	35-40	China Jiangsu	Jiangsu Family Planning Research Institute	12	Psoriasis	NR	MT	9.28; 105.06; 402.88	Y-WS3-98	Y-HPLC
Ji et al. (1998)	CCT	**Group A:** 35.4 [7.3]; **Group B:** 34.4 [9.4]	China Jiangsu	Nanjing General Hospital of Nanjing Military Region	**Group A:** 19; **Group B:** 20	Kidney transplantation	NR	MT	9.28; 105.06; 402.88	Y-WS3-98	Y-HPLC
Zhou et al. (1999)	CCT	**Group A:** 46.5 [11.1]; **Group B:** 49.8 [11.7]; **Group C-G:** NC; **Group H:** ≤40; **Group I:** 41–50; **Group J:** 51–60; **Group K:** 61-70; **Group L:** >70	China Shanghai	Department of Rheumatology, Shanghai Guanghua Hospital of Integrated Chinese and Western Medicine	**Group A:** 20; **Group B:** 50; **Group C:** 40; **Group D:** 47; **Group E:** 36; **Group F:** 27; **Group G:** 4; **Group H:** 15; **Group I:** 26; **Group J:** 18; **Group K:** 30; **Group L:** 7	Rheumatoid arthritis	75.71% (159)	MT	9.28; 105.06; 402.88	Y-WS3-98	Y-HPLC
Li (2000)	Single-arm	**Case A:** 22; **Case B:** 41	China Beijing	The Third Hospital of Beijing Medical University	2	**Case A:** Pyoderma; **Case B:** Gangrenosum	100% (Luo et al., 2019)	NR	NR	N	N
Wu et al. (2001)	RCT	58.6 [2.6]	China Shanghai	Department of Rheumatology, Shanghai Guanghua Hospital of Integrated Chinese and Western Medicine	35	Rheumatoid arthritis	22.86% (Wang et al., 2018)	MT	9.28; 105.06; 402.88	Y-WS3-98	Y-HPLC
Ji et al. (2002)	RCT	40.16 [18.22]	China Shanxi	NR	39	Rheumatoid arthritis	NR	NR	NR	N	N
Jin et al. (2003)	RCT	9.4 [2.2]	China Jiangsu	Department of Pediatrics, Affiliated Hospital of Nanjing University of TCM	31	Purpura nephritis	0%	MT	9.28; 105.06; 402.88	Y-WS3-98	Y-HPLC
Gao et al. (2004)	CCT	44.9	China Shanghai	Renji Hospital	65	Uterine leiomyoma	35.40% (Gao et al., 2004)	MT	9.28; 105.06; 402.88	Y-WS3-98	Y-HPLC
Wang et al. (2004)	CCT	NR	China Jiangsu	Nanjing General Hospital of Nanjing Military Region	15	Graves ophthalmopathy	6.67% (Reno et al., 2015)	NR	NR	N	N
Zhou et al. (2004)	CCT	10.7	China Hubei	Department of Pediatrics, Tongji Hospital, Tongji Medical College, Huazhong University of Science and Technology	51	Henoch- schonlein purpura Nephritis	NR	HS	10.13; 102.67; 412.88	Y-WS3-98	Y-HPLC
Zhou et al. (2004)	CCT	38.3 [16.7]	China Jiangsu	NR	55	Rheumatoid arthritis	49.09% (Lin and Qi, 2005)	NR	NR	N	N
Lin and Qi (2005)	RCT	43.8 [1.65]	China Shandong	NR	47	Erosive oral lichen planus	NR	FH	9.78; 92.46; 398.45	Y-WS3-98	Y-HPLC
Yu et al. (2005)	RCT	41.52 [10.48]	China Shanxi	NR	40	Rheumatoid arthritis	38.00% (Zhang et al., 1994)	XL	10.11; 94.29; 381.23	Y-WS3-98	Y-HPLC
Lei et al. (2006)	RCT	35.0 [12.1]	China Shanghai	Department of Endocrinology, Tongji Hospital	40	Delayed autoimmune diabetes	NR	FH	9.78; 92.46; 398.45	Y-WS3-98	Y-HPLC
Li et al. (2006)	RCT	52.04 [9.30]	China Zhejiang	Zhejiang Provincal Hospital of TCM	60	Rheumatoid arthritis	48.10% (Lei et al., 2006)	BKKY	11.92; 98.52; 396.36	Y-WS3-98	Y-HPLC
Zou and Xiong (2006)	Single-arm	NR	China Jiangsu	The Affiliated Hospital of Nanjing University of TCM	22	Chronic nephritis with persistent proteinuria	31.82% (Zhou et al., 2018)	NR	NR	N	N
Ren et al. (2007)	Single-arm	35.9 [10.9]	China Jiangsu	Multicenter Controlled Study	20	Active Crohn’s disease	33.33% (Zhou et al., 2018)	MT	9.28; 105.06; 402.88	Y-WS3-98	Y-HPLC
Huang et al. (2008)	RCT	**Group A:** 40.9 [8.1]; **Group B:** 41.7 [8.6]	China Hainan	Department of Nephrology, Hainan People’s Hospital	**Group A:** 15; **Group B:** 15	Kidney transplantation with proteinuria	NR	MT	9.28; 105.06; 402.88	Y-WS3-98	Y-HPLC
Mao et al. (2008)	CCT	**Group A:** 34.9 [5.1]; **Group B:** 36.1 [6.0]	China Zhejiang	Department of Nephrology, Hangzhou Hospital of TCM	**Group A:** 24; **Group B:** 34	Chronic glomerular disease	NR	NT	NR	Y-WS3-98	Y-HPLC
Li et al. (2009)	CCT	**Group A:** 13.1; **Group B:** 11.6	China Beijing	NR	**Group A:** 9; **Group B:** 8	Children Alport syndrome	NR	NR	NR	N	N
Ji et al. (2010)	Single-arm	36.2 [10.9]	China Jiangsu	Jiangsu Province Hospital of TCM	12	Ankylosing spondylitis	0%	DE	9.14; 98.96; 403.28	Y-WS3-98	Y-HPLC
Zhang et al. (2010)	CCT	58.0 [7.9]	China Beijing	Peking Union Medical College Hospital	166	Rheumatoid arthritis	63.25% (105)	NR	NR	N	N
Ren et al. (2011)	RCT	**Group A:** 40.7 [11.9]; **Group B:** 42.8 [13.5]	China Jiangsu	Department of Nephrology, Huaihai Hospital Affiliated to Xuzhou Medical College	**Group A:** 9; **Group B:** 11	Steroid-resistant nephrotic syndrome	NR	MT	9.28; 105.06; 402.88	Y-WS3-98	Y-HPLC
Chen et al. (2012)	Single-arm	90	China Beijing	China-Japan Friendship Hospital	1	Systemic lupus erythematosus	NR	NR	NR	N	N
Ren et al. (2013)	RCT	18–60	China Jiangsu	Jinling Hospital, Nanjing University Medical School	21	Crohn’s disease	36.80% (Wang et al., 2018)	HS	10.13; 102.67; 412.88	Y-WS3-98	Y-HPLC
Ge et al. (2013)	RCT	51.9 [9.8]	China Jiangsu	Reasch Institute of Nephrology, Jinling Hospital, Nanjing University School of Medicine	34	Diabetic nephropathy	38.40% (Ao et al., 1994)	MT	9.28; 105.06; 402.88	Y-WS3-98	Y-HPLC
Lv et al. (2015)	RCT	**Group A:** 51.3 [8.3]; **Group B:** 50.6 [8.6]	China Beijing	9 general hospitals with divisions of rheumatology in China	**Group A:** 69; **Group B:** 69	Rheumatoid arthritis	47.82% (Xi et al., 2017)	DE	9.14; 98.96; 403.28	Y-WS3-98	Y-HPLC
Sheng et al. (2013)	RCT	37.5 [11.2]	China Jiangsu	6 hospitals in China	99	Chronic primary glomerular disease	NR	MT	9.28; 105.06; 402.88	Y-WS3-98	Y-HPLC
Zhang et al. (2013)	RCT	18–68	China Shanghai	Shanghai Dermatology Hospital	45	Refractory chronic urticaria	11.11% (Liu et al., 2018)	HS	10.13; 102.67; 412.88	Y-WS3-98	Y-HPLC
He et al. (2014)	RCT	55.12 [8.02]	China Guangdong	Xiangmi Lake Rheumatism Branch of Shenzhen Futian District People’s Hospital	24	Rheumatoid arthritis	NR	MT	9.28; 105.06; 402.88	Y-WS3-98	Y-HPLC
Xu et al. (2014)	RCT	33.0 [11.5]	China Zhejiang	Department of ophthalmology, Jinhua Hospital of TCM	32	Graves ophthalmopathy	NR	MT	9.28; 105.06; 402.88	Y-WS3-98	Y-HPLC
Liu et al. (2015)	CCT	48.82 [6.80]	China Zhejiang	The First Affiliated Hospital, College of Medicine, Zhejiang University	23	Idiopathic membranous nephropathy	26.0% (Zhang et al., 2018)	DE	9.14; 98.96; 403.28	Y-WS3-98	Y-HPLC
Jiang et al. (2015)	RCT	**Group A:** 46.72 [10.71]; **Group B:** 45.48 [11.74]	China Beijing	8 authorized rheumatology departments in general hospitals in China	**Group A:** 46; **Group B:** 51	Rheumatoid arthritis	25.77% (Zhou et al., 2004)	MT	9.28; 105.06; 402.88	Y-WS3-98	Y-HPLC
Sun et al. (2015)	RCT	**Group A:** 31 [8]; **Group B:** 30 [13]	China Jiangsu	The Inflammatory Bowel Disease Center of Jinling Hospital	**Group A:** 68; **Group B:** 71	Idiopathic refractory Nephrotic syndrome	43.17% (Tian et al., 2012)	MT	9.28; 105.06; 402.88	Y-WS3-98	Y-HPLC
Zhu et al. (2015)	RCT	33.2 [11.0]	China Jiangsu	The Inflammatory Bowel Disease Center of Jinling Hospital	45	Crohn’s disease	48.89% (Jin et al., 2003)	MT	9.28; 105.06; 402.88	Y-WS3-98	Y-HPLC
Zhou et al. (2016)	RCT	30.5 [3.6]	China Zhejiang	4 hospitals in China	69	Endometriosis	NR	MT	9.28; 105.06; 402.88	Y-WS3-98	Y-HPLC
Zhu et al. (2016)	RCT	8.32 [3.15]	China Zhejiang	Li Huili East Hospital	52	Purpura nephritis	3.85% (Luo et al., 2019)	MT	9.28; 105.06; 402.88	Y-WS3-98	Y-HPLC
Li et al. (2017)	Single-arm	57	China Beijing	Peking Union Medical College Hospital	1	Synovitis	0%	NR	NR	N	N
Wang et al. (2017)	CCT	**Group A:** 32.5 [11.6]; **Group B:** 33.6 [11.8]	China Jiangsu	Yancheng Third People’s Hospital	**Group A:** 16; **Group B:** 18	IgA nephropathy	2.94% (Reno et al., 2015)	MT	9.28; 105.06; 402.88	Y-WS3-98	Y-HPLC
Shang et al. (2018)	CCT	49.43 [11.89]	China Beijing	Chinese PLA General Hospital	21	Idiopathic membranous nephropathy	28.57% (Zhang et al., 2018)	NR	NR	N	N
Wang (2018)	RCT	**Group A:** 58 [3.8]; **Group B:** 59 [4.2]	China Tianjin	Department of Endocrinology, Tianjin First Center Hopital	**Group A:** 20; **Group B:** 20	Diabetic nephropathy	2.5% (Reno et al., 2015)	MT	9.28; 105.06; 402.88	Y-WS3-98	Y-HPLC
Wu et al. (2015)	RCT	42.0 [12.0]	China Beijing	Peking Union Medical College Hospital	58	Psoriasis	43.60% (Zhou et al., 2004)	NR	NR	N	N
Zhou et al. (2018)	RCT	18–65	USA	Department of Rheumatology and Clinical Immunology, Peking Union Medical College Hospital	**Group A:** 69; **Group B:** 69	Rheumatoid arthritis	49.28% (Li et al., 2014)	NR	NR	N	N

NR, not reported; CCT, controlled clinical trial; RCT, randomized controlled trial; TCM, traditional Chinese medicine; AEs, adverse events; TZ, Jiangsu Taizhou Pharmaceutical Co., Ltd; HQ, Hongqi Pharmaceutical Factory of Shanghai Medical University; HS, Hubei Huangshi Feiyun Pharmaceutical Co., Ltd; XL, Hunan Xieli Pharmaceutical Co., Ltd; FH, Shanghai Fudan Fuhua Pharmaceutical Co., Ltd; BKKY, Zhejiang Prokokangyu Pharmaceutical Co., Ltd; NT, Jiangsu Nantong Pharmaceutical Co., Ltd; DE, Zhejiang, Deend Pharmaceutical Co., Ltd; MT, Jiangsu Meitong Pharmaceutical Co., Ltd; Y-WS3-98, Yes-State food and drug administration of the People’s Republic of China; The 17th volume of Chinese Traditional Medicine preparations standard, No. WS3-B-3350-98; Y-HPLC, Yes-High performance liquid chromatography; TwHF, Tripterygium wilfordii Hook F.

The overall incidence of AEs presented heterogeneity was as high as 30.75% [95% confidence interval (CI) (21.18–40.33), *I* ^2^ = 97%] (**Supplementary Figure 1**). The results of meta-regression of current AEs among patients treated with TWP revealed that high-quality studies, combined medications, medication course, drug dosage, pharmaceutical manufacturers, and organ-specific AEs (*P* < 0.05) significantly affected heterogeneity (**Table 2**). However, studies of lower quality were associated with higher *P* value, suggesting that studies with multivariate adjustment retained some residual confounding observations.

**Table 2:** Potential prespecified sources of heterogeneity among the studies reporting AEs associated with TWP treatment.

Prespecified source of heterogeneity	Number of studies	Stratified random-effects meta-analysis, OR (95% CI)	Meta-regression *P*-value for heterogeneity
**Study quality**			
Lower (0–4)	5	4.98 [−1.79, 11.76]	0.1493
High (5–7)	24	1.60 [1.12, 2.08]	<0.0001
**Combined medication or not**			
with CM	14	1.92 [1.00, 2.84]	<0.0001
without CM	15	1.04 [0.40, 1.68]	0.0014
**Courses of medication**			
≤3 months	13	1.57 [1.13, 2.01]	<0.0001
>3 months	15	1.42 [0.51, 2.33]	0.0022
Dosage of TWP			
<1 OR >1.5 mg/kg/d	14	1.88 [1.42, 2.35]	<0.0001
[1,1.5] mg/kg/d	15	2.14 [1.45, 2.83]	<0.0001
**Pharmaceutical manufacturer**			
TZ	10	1.43 [0.47, 2.39]	0.0036
Others	9	1.25 [0.56, 1.93]	0.0004
**Organ-special AEs**			
Intestinal toxicity	22	0.87 [0.64, 1.10]	<0.0001
Reproductive toxicity	15	0.61 [0.32, 1.02]	0.0032
Hepatotoxicity	22	0.87 [0.47, 1.07]	<0.0001
Nephrotoxicity	6	1.80 [0.03, 3.57]	0.0459
Hematotoxicity	15	0.80 [0.40, 1.20]	<0.0001
Cutaneous toxicity	8	0.81 [0.41, 1.20]	<0.0001
Other damages	11	0.94 [0.59, 1.28]	<0.0001

TWP, Tripterygium Wilfordii tgpolyglycoside; AEs, adverse events; CI, confidence interval; OR, odds ratio; SA: single-arm trials; CM, combined medication; TZ, Jiangsu Taizhou Pharmaceutical Co., Ltd.

### Global Incidence of AEs Triggered by TWP

Twenty-nine studies reported a specific number of AEs after using TWP (1563 patients with 578 events). The incidence of AEs in any grades and severe grade after TWP treatment was 30.75% [95% CI (21.18–40.33), *I* ^2^ = 97%] (**Supplementary Figure 1**) and 4.68% [95% CI (0.00–12.72), *I* ^2^ = 53%), respectively (**Supplementary Figure 2**). Fifteen trials involved treatment with only TWP (1721 patients with 427 events), and the incidence of AEs in these trials was 40.14% [95% CI (29.51–51.58), *I* ^2^ = 89%]. Sixteen trials involved treatment with TWP plus combined drugs (574 patients with 147 events), and the incidence was 22.69% [95% CI (15.04–32.75), *I* ^2^ = 77%) (**Supplementary Figure 3**).

In 36 trials, the TWP-induced AEs usually disappeared after discontinuing the medicine. Only one (with one patient) trial (Li, 2000) showed that the AEs improved at the end of the treatment and disappeared in 3 weeks after withdrawal. However, there was no improvement in nephrotoxicity after 3 months of drug withdrawal in one trial (with one patient) (Tao et al., 1995).

In 32 trials, medical countermeasures were provided to alleviate the AEs, and continuous TWP treatment was discontinued in 15 clinical trials (Ao et al., 1994; Zhang et al., 1994; Zhou et al., 1999; Li, 2000; Gao et al., 2004; Zhou et al., 2004; Li et al., 2006; Zou and Xiong, 2006; Li et al., 2009; Ge et al., 2013; Sheng et al., 2013; Zhang et al., 2013; He et al., 2014; Lv et al., 2015; Zhu et al., 2015). In 7 of these 15 trials (with 40 patients), (Ao et al., 1994; Zhou et al., 1999; Li, 2000; Wang et al., 2004; Zou and Xiong, 2006; Zhang et al., 2013; Zhu et al., 2015) the AEs disappeared after discontinuing TWP treatment, although they did not specifically discuss the conditions of the patients after drug withdrawal; only one trial (Tao et al., 1995) showed no improvement after 3 months of drug discontinuation. Treatment plan was not discontinued or changed in 6 of 46 studies (with 54 patients) as the AEs were mild and well controlled (28, 32, 47, 55, 56, 57. Furthermore, symptomatic treatment was provided in 6 of 46 trials, (Wu et al., 2001; Zhou et al., 2004; Mao et al., 2008; Ren et al., 2011; Sheng et al., 2013; Zhou et al., 2016) reduced dosage was provided in 3 trials, (Ji et al., 1998; Huang et al., 2008; Zhang et al., 2010) and 1 (Zhang et al., 1994) trial involved change in the time of medicine administration after meals.

### Course of Medication and Combined Medication

In 46 studies, 39.1% (18/46) of the diagnoses were related to urinary system diseases, 23.9% (11/46) to RA, 10.9% (5/46) to digestive system diseases, and 8.7% (4/46) to skin diseases (including 2 cases of psoriasis, 1 case of chronic urticaria, and 1 case of gangrenous pyoderma). There were two cases of Graves’ eye disease, one case of uterine fibroids, one case of endometriosis, one case of oral lichen planus, one case of ankylosing spondylitis, one case of lupus erythematosus, and one case of periostitis. The details are shown in **Figure 2**.

The incidence of AEs of all grades in patients without any combined medication was 40.16% [95% CI (23.51–56.80), *I* ^2^ = 98%], whereas that with combined medication was 22.69% [95% CI (15.04–32.75), *I* ^2^ = 77%]. The incidence of AEs was 33.86% [95% CI (21.39–49.06), *I* ^2^ = 76%] in patients (with 104 patients) treated with a combination of TWP and methotrexate (MTX), 21.81% [95% CI (8.18–46.62), *I* ^2^ = 28%] in patients (with 8 patients) treated with a combination of glucocorticoid and TWP, 30.48% [95% CI (18.07–46.58), *I* ^2^ = 46%] in patients (with 23 patients) treated with a combination of TWP and non-steroidal anti-inflammatory drugs (NSAIDs), 24.38% [95% CI (5.67–63.37), *I* ^2^ = 50%] in patients (with 26 patients) treated with a combination of TWP and antibiotic, 7.78% [95% CI (1.00–41.36), *I* ^2^ = 83%] in patients (with 9 patients) treated with combination of TWP and CHM, and 13.10% [95% CI (4.73–31.40), *I* ^2^ = 63%, with 15 patients] in patients treated with a combination of TWP and symptomatic medication. The detailed information on combined medication is shown in **Table 3** and **Supplementary Figure 4**.

**Table 3:** Effect of estimates of AEs in the 46 studies included in this meta-analysis.

Trials	Any-grade AEs				
	Events	Total	Incidence	*I* ^2^	P value
**3.1 AEs with TWP combined with multiple drug treatments**					
**3.1.1 Combined with methotrexate**					
Wu et al. (2001)	8	35	22.86 [10.42, 40.14]		
Ji et al. (2010)	0	12	0.00 [0.00, 26.47]		
Lv et al. (2015)	34	166	49.28 [37.02, 61.59]		
Jiang et al. (2015)	25	69	25.77 [17.43, 35.65]		
Li et al. (2017)	0	97	0.00 [0.00, 97.5]		
Zhou et al. (2018)	35	1	50.73 [38.41, 62.98]		
Meta-analysis			33.86 [21.39, 49.06]	76%	<0.01
**3.1.2 Combined with glucocticoid**					
Li (2000)	1	2	50.00 [1.26, 98.74]		
Wang et al. (2004)	1	15	6.67 [0.17, 31.95]		
Liu et al. (2015)	6	23	26.09 [10.23, 48.41]		
Meta-analysis			21.81 [8.18, 46.62]	28%	0.25
**3.1.3 Combined with NSAIDs**					
Wu et al. (2001)	8	35	22.86 [10.42, 40.14]		
Yu-wen et al. (2005)	15	40	37.50 [22.73, 54.20]		
Meta-analysis			30.48 [18.07, 46.58]	46%	0.17
**3.1.4 Combined with antibiotic**					
Li (2000)	1	1	1.00 [2.50, 1.00]		
Ji et al. (2010)	0	12	0.00 [0.00, 26.47]		
Jiang et al. (2015)	25	97	25.77 [17.43, 35.65]		
Meta-analysis			24.38 [5.57, 63.37]	50%	<0.01
**3.1.5 Combined with Chinese medicine**					
Jin et al. (2003)	0	31	0.00 [0.00, 11.22]		
Zou and Xiong (2006)	7	22	31.82 [13.87, 54.87]		
Zhu et al. (2016)	2	52	3.85 [0.47, 13.21]		
Meta-analysis			7.78 [1.00, 41.36]	83%	<0.01
**3.1.6 Combined with symptomatic medication**					
Li (2000)	2	2	100.00 [15.81, 100.00]		
Wang et al. (2004)	1	15	6.67 [0.17, 31.95]		
Ji et al. (2010)	0	12	0.00 [0.00, 26.47]		
Zhang et al. (2013)	5	45	11.11 [3.71, 24.05]		
Shang et al. (2018)	6	21	28.57 [11.28,52.18]		
Wang (2018)	1	40	2.50 [0.06, 13.16]		
Meta-analysis			13.10 [4.73, 31.40]	63%	0.02
**3.2 AEs with TWP combined with different dosages**					
**3.2.1 < 0.5 mg·kg/d**					
Zou and Xiong (2006) 0.3 mg·kg/d	7	22	31.82 [13.87, 54.87]		
**3.2.2 0.5 mg·kg/d**					
Zhou et al. (1999) 0.5 mg·kg/d	32	90	35.56 [25.74, 46.35]		
Zhou et al. (1999) 0.6 mg·kg/d	38	83	45.78 [34.79, 57.08]		
Li (2000) 0.5 mg·kg/d	1	1	100 [2.50, 100.00]		
Wu et al. (2001) 0.5 mg·kg/d	8	35	22.86 [10.42, 40.14]		
Li et al. (2006) 0.5 mg·kg/d	29	60	48.33 [35.23, 61.61]		
Jiang et al. (2015) 0.5 mg·kg/d	25	97	25.77 [17.43, 35.65]		
Meta-analysis			36.47 [27.48, 46.51]	67%	0.01
**3.2.3 0.75 mg·kg/d**					
Zhou et al. (1999) 0.7 mg·kg/d	13	31	41.94 [24.55, 60.92]		
Zhou et al. (1999) 0.8 mg·kg/d	29	41	70.73 [54.46, 83.87]		
Zhou et al. (1999) 0.9 mg·kg/d	39	48	81.25 [67.37, 91.05]		
Liu et al. (2015) [0.5,1] mg·kg/d	6	23	26.09 [10.23, 48.41]		
Li et al. (2017) [0.5,1] mg·kg/d	0	1	0.00 [0.00, 97.50]		
Meta-analysis			54.34 [30.46, 76.37]	84%	<0.01
**3.2.4 1 mg·kg/d**					
Zhou et al. (1999) 1 mg·kg/d	18	27	66.67 [46.04, 83.48]		
Li (2000) 1 mg·kg/d	1	1	100.00 [2.50, 100.00]		
Jin et al. (2003) 1 mg·kg/d	0	31	0.00 [0.00, 11.22]		
Yu-wen et al. (2005) 1 mg·kg/d	15	40	37.50 [22.73, 54.20]		
Ji et al. (2010) 1 mg·kg/d	0	12	0.00 [0.00, 26.47]		
Ren et al. (2013) 1 mg·kg/d	8	21	38.10 [18.11, 61.57]		
Lv et al. (2015) 1 mg·kg/d	66	138	47.83 [39.26, 56.49]		
Zhang et al. (2013) 1 mg·kg/d	5	45	11.11 [3.71, 24.05]		
Shang et al. (2018) 1 mg·kg/d	6	21	28.57 [11.28, 52.18]		
Wang (2018) 1 mg·kg/d	1	40	2.50 [0.06, 13.16]		
Wu et al. (2015) 1 mg·kg/d	25	58	43.10 [30.16, 56.77]		
Zhou et al. (2018) 1 mg·kg/d	68	138	49.28 [40.67, 57.92]		
Meta-analysis			33.75 [23.92, 45.22]	78%	<0.01
**3.2.5 1.5 mg·kg/d**					
Zhang et al. (1994) 1.5 mg·kg/d	3	30	10.00 [2.11, 26.53]		
Zhu et al. (2015) 1.5 mg·kg/d	22	45	48.89 [33.70, 64.23]		
Zhu et al. (2016) 1.5 mg·kg/d	2	52	3.85 [0.47, 13.21]		
Meta-analysis			14.86 [2.24, 57.10]	78%	<0.01
**3.2.6 > 1.5 mg·kg/d**					
Gao et al. (2004) 2 mg·kg/d	23	65	35.39 [23.92, 48.23]		
Sun et al. (2015) [1.5,2] mg·kg/d	40	139	28.78 [21.42,37.06]		
Meta-analysis			30.96 [24.98, 37.65]	0%	0.34
**3.3 Course treatment of AEs with TWP**					
**3.3.1 1 month**					
Jin et al. (2003) 1 month	0	31	10.00 [2.11, 26.53]		
Yu-wen et al. (2005) 6 weeks	15	40	38.10 [18.11, 61.57]		
Ji et al. (2010) 6 weeks	0	12	28.78 [21.42, 37.06]		
Zhang et al. (2013) 1 month	5	45	48.89 [33.70, 64.23]		
Meta-analysis			31.22 [18.61, 47.41]	76%	<0.01
**3.3.2 2 months**					
Li et al. (2006) 2 months	29	60	48.33 [35.23, 61.61]		
Wu et al. (2015) 2 months	25	58	43.10 [30.16, 56.77]		
Meta-analysis			45.78 [37.00, 54.82]	0	0.57
**3.3.3 3 months**					
Ao et al. (1994) 3 months	4	87	4.60 [1.27, 11.36]		
Zhou et al. (1999) 3 months	41	85	48.24 [37.26, 59.34]		
Wu et al. (2001) 3 months	8	35	22.86 [10.42, 40.14]		
Wang et al. (2004) 3 months	1	15	6.67 [0.17, 31.95]		
Zhou et al. (2004) 3 months	27	55	49.10 [35.35, 62.93]		
Zhu et al. (2016) 3 months	2	52	3.85 [0.47, 13.21]		
Meta-analysis			17.73 [7.02, 38.10]	90%	<0.01
**3.3.4 6 months**					
Zhou et al. (1999) >3 months	118	236	50.00 [43.44, 56.56]		
Gao et al. (2004) [3,6]months	23	65	35.39 [23.92, 48.23]		
Ge et al. (2013) 6 months	13	34	38.24 [22.17, 56.44]		
Lv et al. (2015) 6 months	66	138	47.83 [39.26, 56.49]		
Jiang et al. (2015) 6 months	25	97	25.77 [17.43, 35.65]		
Li et al. (2017) 6 months	0	1	0.00 [0.00, 97.50]		
Shang et al. (2018) 6 months	6	21	28.57 [11.28, 52.18]		
Wang (2018) 6 months	1	40	2.50 [0.06, 13.16]		
Zhou et al. (2018) 6 months	68	138	49.28 [40.67, 57.92]		
Meta-analysis			37.79 [29.63, 46.69]	77%	<0.01
**3.3.5 9 months**					
Liu et al. (2015) 9 months	6	23	26.09 [10.23, 48.41]		
**3.3.6 12 months**					
Zhang et al. (1994) 12 months	3	30	10.00 [2.11, 26.53]		
Ren et al. (2013) 12 months	8	21	38.10 [18.11, 61.57]		
Sun et al. (2015) 12 months	40	139	28.78 [21.42, 37.06]		
Zhu et al. (2015) 12 months	22	45	48.89 [33.70, 64.23]		
Meta-analysis			31.22 [18.61, 47.41]	76%	<0.01
**3.4 Medication treatment of AEs with TWP**					
**3.4.1 Meitong Pharmaceutical**					
Ao et al. (1994)	4	87	4.60 [1.27, 11.36]		
Jiang et al. (1994)	12	13	92.31 [64.00, 99.81]		
Zhou et al. (1999)	159	320	49.69 [44.08,55.30]		
Wu et al. (2001)	8	35	22.86 [10.42, 40.14]		
Jin et al. (2003)	0	31	0.00 [0.00,11.22]		
Gao et al. (2004)	23	65	35.39 [23.92, 48.23]		
Ge et al. (2013)	13	34	38.24 [22.17, 56.44]		
Sun et al. (2015)	40	139	28.78 [21.42, 37.06]		
Zhu et al. (2015)	22	45	48.89 [33.70, 64.23]		
Wang et al. (2017)	1	34	2.94 [0.07, 15.33]		
Jiang et al. (2015)	25	97	25.77 [17.43, 35.65]		
Zhu et al. (2016)	2	52	3.85 [0.47, 13.21]		
Wang (2018)	1	40	2.50 [0.06, 13.16]		
Meta-analysis			23.09 [14.74, 34.26]	89%	<0.01
**3.4.2 Zhejiang Deend Pharmaceutical**					
Ji et al. (2010)	0	12	0.00 [0.00, 26.47]		
Lv et al. (2015)	66	138	47.83 [39.26, 56.49]		
Liu et al. (2015)	6	23	26.09 [10.23, 48.41]		
Meta-analysis			29.99 [11.89, 57.63]	75%	0.02
**3.4.3 Huangshi Feijun Pharmaceutical**					
Ren et al. (2013)	8	21	38.10 [18.11, 61.57]		
Zhang et al. (2013)	5	45	11.11 [3.71, 24.05]		
Meta-analysis			21.84 [5.54, 57.12]	83%	0.01
**3.4.4 Others**					
Yu-wen et al. (2005)	15	40	37.50 [22.73, 54.20]		
Li et al. (2006)	29	60	48.33 [35.23, 61.61]		
Zhang et al. (1994)	3	30	10.00 [2.11, 26.53]		
Meta-analysis			31.55 [15.04, 54.54]	81%	<0.01

AEs, adverse events; TWP, Tripterygium wilfordii tgpolyglycoside; NSAIDs, non-steroidal anti-inflammatory drugs.

### Dosage-Dependent Analysis of AEs

Twenty-nine of the 46 trials showed the number and grade of AEs after treatment with specific doses of TWP. The incidence of all types of AEs was 36.47% [95% CI (27.48–46.51), *I* ^2^ = 0%] in patients treated with 0.5 mg/kg/d TWP (6/46 studies, with 133 patients), 54.34% [95% CI (30.46–76.37), *I* ^2^ = 84%] in patients treated with 0.75 mg/kg/d TWP (5/46 studies, with 87 patients), 33.75% [95% CI (23.92–45.22), *I* ^2^ = 87%] in patients treated with 1.0 mg/kg/d TWP (12/46 studies, with 213 patients), 14.86% [95% CI (2.24–57.10), *I* ^2^ = 91%] in patients treated with 1.5 mg/kg/d TWP (3/46 studies, with 27 patients), and 30.96% [95% CI (24.98–37.65), *I* ^2^ = 0%] in patients treated with more than 1.5 mg/kg/d TWP (2/46, with 63 patients). The details are shown in **Table 3** and **Supplementary Figure 5**.

Forty-three of the 46 studies showed the dose frequency in the meta-analysis. We selected 15 of those trials that involved therapeutic doses ranging from 1 to 1.5 mg/kg/d to avoid heterogeneity, and counted the frequency of medicine consumption. However, in most studies (13/15), patients consumed the medicine thrice a day; hence, we could not determine the correlation between medicine consumption frequency and AEs.

### Medication and AEs

Twenty-six of the 46 trials showed the specific number of patients and the treatment duration with TWP. The incidence of AEs of all grades was 11.56% [95% CI (2.92–36.24), *I* ^2^ = 79%] in patients (4/46 studies, with 20 patients) treated with TWP for 1 month, 45.78% [95% CI (37.00–54.82), *I* ^2^ = 0%] in patients (2/46 studies, with 54 patients) treated with TWP for 2 months, 17.73% [95% CI (7.02–38.10), *I* ^2^ = 90%) in patients (6/46 studies, with 83 patients) treated with TWP for 3 months, 37.79% [95% CI (29.63–46.69), *I* ^2^ = 77%] in patients (9/46 studies, with 320 patients) treated with TWP for 6 months, and 31.22% [95% CI (18.61–47.41), *I* ^2^ = 76%] in patients (4/46 studies, with 73 patients) treated with TWP for 12 months. Specific data are shown in **Table 3** and **Supplementary Figure 6**.

### Pharmaceutical Manufacturer

Thirty-five of the 46 studies clearly mentioned the name of the pharmaceutical manufacturer with specific events. The incidence of AEs was 23.01% [95% CI (14.74–34.26), *I* ^2^ = 89%] in patients (13/46, with 310 patients) treated with TWP manufactured by Jiangsu Meitong Pharmaceutical Co., Ltd.; 29.99% [95% CI (11.89–57.63), *I* ^2^ = 75%] in patients (3/46, with 72 patients) treated with TWP manufactured by Zhejiang Deend Pharmaceutical Co., Ltd.; 21.84% [95% CI (5.54–57.12), *I* ^2^ = 83%] in patients (2/46, with 13 patients) treated with TWP manufactured by Huangshi Feiyun Pharmaceutical Co., Ltd.; and 31.55% [95% CI (15.04–54.54), *I* ^2^ = 81%] per the rate provided by other pharmaceutical companies. The statistical details are shown in **Table 3** and **Supplementary Figure 7**.

### Organ-Specific AEs

**Figure 4:**
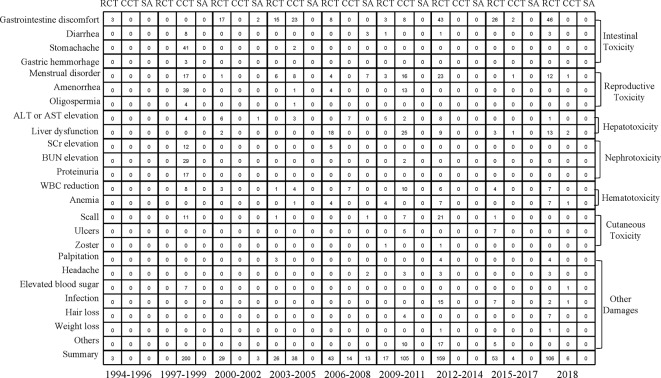
Mapping of seven organ-specific adverse events of study type between 1994 and 2018. RCT, randomized controlled trial; CCT, controlled clinical trial; SA, single-arm trial.

We analyzed the incidence of AEs associated with intestinal toxicity, reproductive toxicity, hepatotoxicity, nephrotoxicity, hematotoxicity, and cutaneous toxicity during drug use. The most common AE of all grades caused by TWP was intestinal toxicity, followed by reproductive toxicity. The detailed information regarding organ-specific AEs is shown in **Figure 4** and **Supplementary Figure 8**.

### Intestinal Toxicity

The most common digestive AE during treatment with TWP was gastrointestinal discomfort, with an incidence of 13.88% [95% CI (9.12–20.56), *I* ^2^ = 85%). In addition, gastric hemorrhage was a serious symptom in the digestive system, although the probability of occurrence was low (**Supplementary Figure 9**).

### Reproductive Toxicity

Amenorrhea was the most frequent AE in patients treated with TWP, and its incidence was 10.16% [95% CI (5.54–18.16), *I* ^2^ = 78%). No serious AE occurred in the reproductive system (**Supplementary Figure 10**).

### Hepatotoxicity

The incidence of increased levels of aspartate aminotransferase (AST) or alanine aminotransferase (ALT) was 6.83% [95% CI (3.87–11.77), *I* ^2^ = 62%] without any serious hepatotoxic AEs (**Supplementary Figure 11**).

### Nephrotoxicity

The most frequent urinary system AE was proteinuria, with an incidence of 13.57% [95% CI (6.87–25.07), *I* ^2^ = 54%]. The incidence of elevated serum creatinine (SCr) or blood urea nitrogen (BUN) levels ranged from 6% to 8% after TWP treatment (**Supplementary Figure 12**).

### Hematotoxicity

The most common hematotoxic AE arising during the use of TWP was leukopenia, which might develop into a life-threatening severe AE. Fortunately, it was usually not critical and could be corrected by timely medical intervention. The incidence of hematotoxicity was 5.66% [95% CI (4.27–7.47), *I* ^2^ = 25%], and that of leukopenia was 5.74% [95% CI (3.94–8.31), *I* ^2^ = 38%) (**Supplementary Figure 13**).

### Cutaneous Toxicity

The incidence of all grades of skin system AEs was less than 5%. The general AEs of the skin included scall, ulcers, and herpes zoster, which were not of severe grade (**Supplementary Figure 14**).

### Other Damages

The incidences of other damages caused by TWP, such as palpitation, headache, elevated blood sugar, and hair loss, were all <5%. The incidence of other damages was 4.43% [95% CI (2.92–6.67), *I* ^2^ = 72%] at a low rate of AEs (**Supplementary Figure 15**).

### Quality Assessment, Heterogeneity, and Publication Bias

The Cochrane Collaboration’s tool was used to evaluate the RCTs’ methodological qualities in the meta-analysis, and the NOS was used for CCTs and single-arm trials. The overall risk of bias was assessed as low risk. Hence, the quality of the studies included was satisfactory (**Supplementary Tables 1 and 2**).

The meta-regression analysis revealed that high-quality studies, combined medications, medication course, drug dosage, pharmaceutical manufacturer, and organ-specific AEs (P < 0.05) had a significant effect on heterogeneity in the meta-analysis (**Table 2**).

The funnel plot and Egger’s test were used for detecting publication bias, which showed an asymmetric distribution of trials along the side of the funnel (**Supplementary Figure 16**). TWP was used in the different groups of the included studies. Owing to the different variable properties in each article, the results of the meta-analysis may have been affected by interference from unrelated variables. Egger’s test showed that *P* = 0.02364 (< 0.05) indicated the presence of publication bias (**Supplementary Figure 17**).

## Discussion

The purpose of this study was to review the safety of TWP and provide a reference for TwHF preparations. It must be noted that the conclusions of this study can only serve as a reference for AEs caused by preparation types besides TWP, as the main toxicity component triptolide in TwHF is removed in TWP (Tian et al., 2012). In addition, owing to the different forms of articles included in this meta-analysis, the amount of information contained in each article differs. As much as possible, we performed subgroup analysis for studies with the same characteristics, which may have led to differences in total study counts and subgroup study counts. However, incorporating different types of articles is also one of the major strengths of this meta-analysis. A study (which included 428 trials) published in 2016 reported that the incidence of all grades of AEs caused by TwHF preparations was 26.7% [95% CI (24.8–28.8)]. The results of that systematic review were similar to our findings, but it included all types of TwHF preparations and 94% (402/428) of the included trials were from Chinese databases (Zhang et al., 2018). The use of all types of TwHF preparations might increase statistical heterogeneity, and literatures of low quality might lead to low quality of methodology. In this analysis, we excluded other types of TwHF preparations, and the articles were all from English databases to ensure the quality of the outcomes.

We included 46 trials with 2437 patients in this study. The incidence of AEs of all grades in patients treated with TWP was 30.75% (**Supplementary Figure 1**). In comparison, the incidence of severe-grade AEs was only 4.68% (**Supplementary Figure 2**). This indicated that the incidence of severe grade AEs caused by TWP was fairly low and that the drug was safe to use. We also observed that the incidence of AEs caused by treatments with TWP without any combination medication was higher than that of AEs caused by TWP treatment combined with other therapeutic methods (**Supplementary Figure 3**). This indicated that TWP had relatively high toxicity, and hence, AEs could be triggered without combining TWP with other drugs. Next, we conducted a subgroup analysis of the trials according to the combinations of different interventions with TWP and observed the highest incidence in the TWP plus MTX group, followed by the TWP plus NSAIDs and TWP plus antibiotics groups. The group with the lowest incidence was TWP plus Chinese medicine (**Table 3**). Interestingly, the incidence of AEs caused by TWP with all types of combined medications was lower than that caused by TWP without any combination. This indicated that combination with other drugs may weaken the toxicity caused by TWP and reduce the occurrence of AEs (**Supplementary Figure 4**). A previous study (Cao et al., 2018) performed a similar subgroup analysis between TwHF monotherapy and combination therapy. Many of the symptoms, including leukopenia, gastrointestinal reactions, irregular menstruation, and abnormal liver function, were observed in patients on TwHF without any combinations at a higher frequency than in patients on combined therapy. However, this previous study included 36 RCTs that were reported in 2015. With the emergence of more evidence, systematic reviews should be updated in a timely manner. In our meta-analysis, high-quality RCTs, CCTs, and single-arm trials were added, and a set of rigid inclusion and exclusion criteria was used. The results proved that AEs observed after treatment with combined medications were related to the original toxicity of the drugs.

Furthermore, we observed that almost all AEs occurred during the duration of the medication. Meanwhile, AEs could be alleviated by reducing the dosage of the drugs or by changing the medicine consumption habit, such as consuming the medicine after meals. The AE grade was reduced after 3 months of drug withdrawal. This showed that the incidence of AEs decreased in patients treated with TWP when the drug was completely metabolized because of the recovery of bodily functions. However, the results might be related to case loss. The recommended dosage of TWP was 1−1.5 mg/kg/d, and we observed that the incidence of AEs with this dosage was 29.01% [95% CI (20.17–39.79), *I* ^2^ = 82%] (**Supplementary Figure 18**), which was lower than that in any other dosage groups. This indicated that either higher or lower dosage may lead to more serious AEs. In addition, the official recommended usage time is 3 months. We also observed an increase in AE incidence after more than 3 months of use (**Supplementary Figure 19**). Thus, TwHF should be used in moderation and for a short term.

We intended to statistically determine the relationship of age and gender with TWP application, although it could not be completed due to the lack of information. A study (Liu et al., 2010) showed that AEs caused by TwHF were more frequent in females than in males. Furthermore, toxicokinetic data indicated that TWP has gender-related differences in drug accumulation and toxicities, and that females are more sensitive to TWP toxicity than males (Liu et al., 2015). These results suggest that women should consider a protective agent for target organs when using TwHF. Owing to the effects of TWP on female menstruation and male sperm production, the most vulnerable age is the child-bearing age.

**Figure 5:**
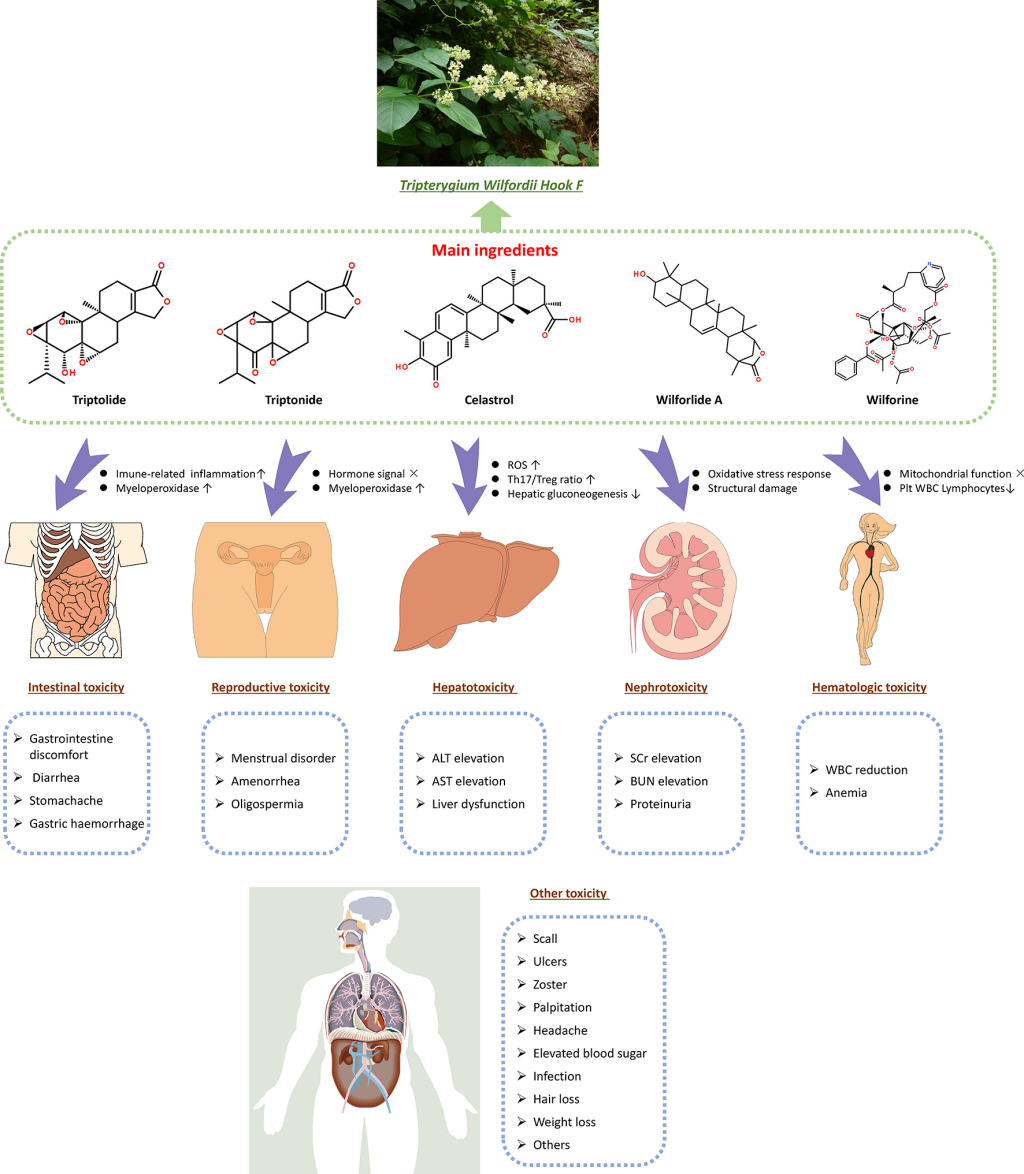
Effective constituents of TwHF (such as triptolide, triptonide, celastrol, and wilforine) are both the active ingredients and toxicity source. TwHF induces toxicity in various target organs. In the digestive system, TwHF causes gastric mucosa irritation and increases the infiltration of inflammatory cells in the gastrointestinal tract. TwHF also disrupts the signaling of sex hormones and myeloperoxidase in reproductive organs, causes oxidative stress, increases the ratio of Th17/Treg, and suppresses hepatic gluconeogenesis. In the kidneys, TwHF decreases the activities of renal SOD and GSH and increases the level of MDA and BUN, resulting in serious oxidative stress and structural damage. TwHF can affect mitochondrial function and reduce the number of platelets, WBC, and lymphocytes. TwHF, *Tripterygium wilfordii* Hook F; ROS, reactive oxygen species; Plt, blood platelet; WBC, white blood cell; ALT, alanine aminotransferase; AST, aspartate aminotransferase; SCr, serum creatinine; BUN, blood urea nitrogen; SOD, superoxide dismutase; GSH, glutathione; MDA, malondialdehyde; WBC, white blood cell.

**Figure 6:**
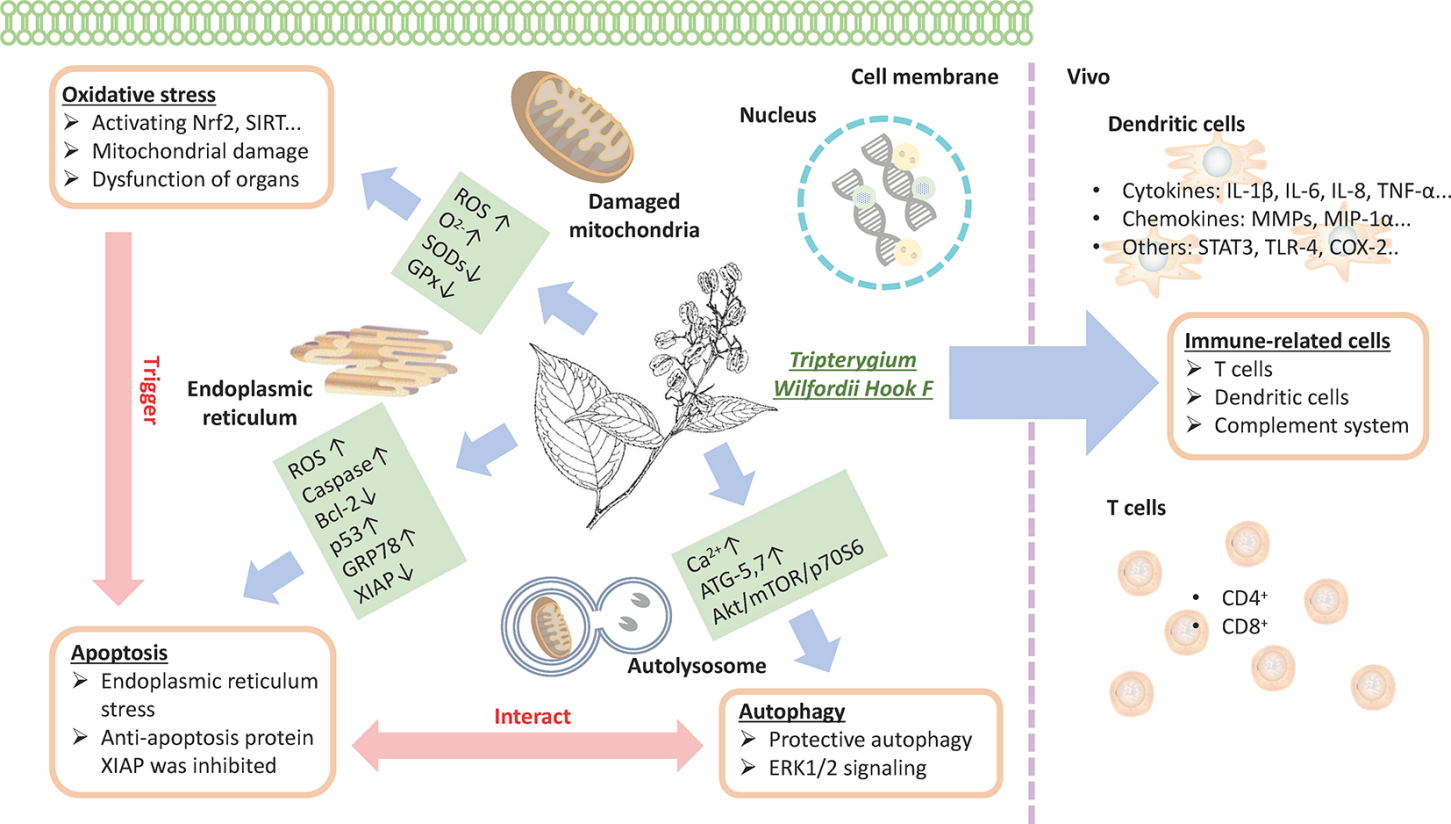
TwHF inhibits the activity of immune cells and prevents T-cell proliferation with the reduction in proinflammatory cytokines and chemokines; TwHF alters intracellular GSH levels, resulting in the overproduction of ROS, leading to mitochondrial damage; TwHF increases the generation of anion superoxide (O^2-^), inhibits the activity of antioxidant enzymes such as SOD and catalase, and induces oxidative stress and dysfunction in organs. Meanwhile, oxidative stress is an important factor in the induction of apoptosis. TwHF treatment activated caspase-3, caspase-8, and caspase-9, and p53 and downregulated Bcl-2 levels in mitochondria-initiated apoptosis. TwHF can increase the expression of GRP78 to inhibit endoplasmic reticulum stress and induce protective autophagy; it also inhibits apoptosis by eliminating dysfunctional mitochondria. TwHF, *Tripterygium wilfordii* Hook F; GSH, glutathione; ROS, reactive oxygen species; SOD, superoxide dismutase.

Previous data show that TwHF has several effective constituents, such as triptolide, triptonide, celastrol, and wilforine, with the most important being triptolide (Fan et al., 2018). These constituents are both the active ingredients and the source of toxicity (Gao et al., 2019). The mechanism of TwHF-induced AEs is complicated, and AEs occur in multiple systems and targets. The effect of TwHF on different systems is shown in **Figure 5**. In addition, the immunoregulatory effects of TwHF are mainly directed to immune cells, immune molecules, and cellular signaling. TwHF exerts multidimensional regulatory activities on T cells and dendritic cells *in vivo* (Xi et al., 2017) (**Figure 6**).

In addition, the quality of a CHM is closely related to the quality of its active ingredients, which is important for both quality evaluation and control. The standard for the extraction of TWP in the market often considers wilforine as the main evaluation index, which is proposed as a biologically active and toxic component of TwHF (Xi et al., 2017). However, TwHF preparations produced by different manufacturers vary in chemical composition, which is consistent with clinical observations (Wang et al., 2016). To overcome this problem, we analyzed the drug produced by different pharmaceutical manufacturers (**Table 3**, **Supplementary Figure 7**) and observed that the heterogeneity between groups was large. This might be due to the lack of information and the fact that all the pharmaceutical companies were from China.

TWP is used in the treatment of various diseases, although it induces a wide spectrum of AEs. In the included trials, the most common AE was intestinal toxicity (**Supplementary Figure 9**), followed by reproductive toxicity (**Supplementary Figure 10**). To summarize, the main AEs that appeared in the included trials were as follows: (i) gastrointestinal tract stimulation, (ii) damage to the reproductive system, (iii) liver or kidney damage, (iv) AEs of skin mucosa, (v) leukopenia, (vi) induced arrhythmia, and (vii) infection or acute poisoning.

In summary, the toxicity of TwHF depends on the dose and time of administration. Notably, both high/short-term doses and low/long-term doses cause AEs (Li et al., 2014). As each individual reacts differently to TWP, which may affect the accuracy of the meta-analysis, we used meta-regression to analyze and divide the subgroups to identify the sources of heterogeneity in this meta-analysis. As TwHF preparations have a prominent anti-inflammatory effect and are clinically irreplaceable, (Yao et al., 2010) we suggest that the clinical dosage of TwHF should be strictly controlled and its usage should be stringently monitored. Furthermore, toxicological studies on TwHF should be conducted to ensure the safety of TwHF preparations.

Our study has some limitations. First, some of the included studies were not RCTs, which was the main source of publication bias. Second, some key information was missing from some of the included studies; for instance, the number of certain specific AEs was not mentioned, and most studies showed the total number of AEs. Furthermore, the clinical environment was highly variable, and hence, the use of the random-effects models was important for controlling variability. Therefore, control variables were used to ensure the quality of the meta-analysis in the subgroup analysis of the drug characteristics and system. Simultaneously, the sample size of different subgroups was small, and some subgroups could not achieve the test effect.

In conclusion, our meta-analysis provided a statistical outline of AEs in patients treated with TWP. The included evidence was entirely from English databases, and the pattern of AE occurrence was organ-specific and related to TwHF.

## Glossary

TWP, *Tripterygium wilfordii* tgpolyglycoside; TwHF, *Tripterygium wilfordii* Hook F; AE, adverse event; CHM, Chinese herbal medicine; RCT, randomized controlled trial; CCT, controlled clinical trial; EMBASE, Excerpta Medica Database; WOS, Web of Science; CTCAE 5.0, the Common Terminology Criteria for Adverse Events 5.0; NOS, Newcastle–Ottawa Scale; CI, confidence interval; SCr, serum creatinine; BUN, blood urea nitrogen; AST, aspartate aminotransferase; ALT, alanine aminotransferase; NSAID, non-steroidal anti-inflammatory drug; MTX, methotrexate; RA, rheumatoid arthritis; ER, endoplasmic reticulum; HPLC, high-performance liquid chromatography.

## Data Availability Statement

The data sets analysed during the current study are available from the corresponding author upon reasonable request.

## Author Contributions

Conceptualization: YR, XL, BL. Data curation: YL, YR. Formal analysis: XL, YingL, YZ; Funding acquisition: BL, LK, XS; Investigation: YR, MX, LL; Methodology: YingL, YiL, SH. Project administration: XL, BL, XC. Resources: JS, YueL, XF. Software: RY, YingL. Supervision: BL, XL; Validation: YZ, LK, XS.

## Funding

This study was supported by the National Key Research and Development Program of China (No. 2018YFC1705301), the NSFC of China (No. 81874470, 81973860), the Young Talent Supporting Program of China Association of Traditional Chinese Medicine [No. CACM-2017-QNRC2-(B05)], the Development Fund for Shanghai Talents (No. 2017047), the Shanghai Development Office of TCM [Nos. ZY(2018-2020)-FWTX-1008, ZY(2018-2020)-CCCX-2004-08, and ZY(2018-2020)-FWTX-4010], and the Scientific Research Project of Yueyang Integrated Traditional Chinese and Western Medicine Hospital affiliated to Shanghai University of traditional Chinese medicine (No. 2017YJ03).

## Conflict of Interest

The authors declare that the research was conducted in the absence of any commercial or financial relationships that could be construed as a potential conflict of interest.
