# Supplementary material for: Adverse Events Associated With Treatment of Tripterygium wilfordii Hook F: A Quantitative Evidence Synthesis
Source: Front Pharmacol. 2019 Nov 6;10:1250. doi: 10.3389/fphar.2019.01250 (PMC6851843; doi:10.3389/fphar.2019.01250)
Supplement: Supplementary file 1 [file DataSheet_1.zip › Supplementary Table 1.DOCX]

**Supplementary Table 1.** The Cochrane Collaboration’s tool for assessing risk of bias of RCT.

|  | Random sequence generation | Allocation concealment | Blinding of participants and personnel | Blinding of outcome assessment | Incomplete outcome data | Selective outcome reporting | Other source of bias |
| --- | --- | --- | --- | --- | --- | --- | --- |
| Zhang X-zhen et al. (1994) | ? | ? | - | - | + | + | ? |
| Wu Y-jun et al. (2001) | ? | ? | - | - | + | + | ? |
| Ji H-wang et al. (2002) | ? | ? | - | - | + | + | ? |
| Jin Z-da et al. (2003) | ? | ? | - | - | + | + | ? |
| Lin L-mei et al. (2005) | + | ? | - | - | + | + | ? |
| Yu-wen Ya et al. (2005) | + | ? | - | - | + | + | ? |
| Lei Tao et al. (2006) | ? | ? | - | - | + | + | ? |
| Li Y-song et al. (2006) | ? | ? | - | - | + | + | ? |
| Huang Yun et al. (2008) | + | ? | - | - | + | + | ? |
| Ren H-qi et al. (2011) | + | ? | - | - | + | + | ? |
| Ren J-nan et al. (2012) | + | ? | + | + | + | + | ? |
| Ge Y-chun et al. (2013) | ? | ? | - | - | + | + | ? |
| Lv Q-wen et al. (2013) | + | ? | + | + | + | + | ? |
| Sheng M-xiao et al. (2013) | + | ? | - | - | + | + | ? |
| Zhang Wei et al. (2013) | ? | ? | - | - | + | + | ? |
| He W-zhen et al. (2014) | + | ? | - | - | + | + | ? |
| Xu J-ping et al. (2014) | ? | ? | - | - | + | + | ? |
| Jiang Miao et al. (2015) | + | + | + | + | + | + | ? |
| Sun Jing et al. (2015) | + | ? | - | - | + | + | ? |
| Zhu W-ming et al. (2015) | + | ? | - | - | + | + | ? |
| Zhou X-hong et al. (2016) | + | ? | - | - | + | + | ? |
| Zhu T-fu et al. (2016) | ? | ? | - | - | + | + | ? |
| Wang Wei et al. (2018) | - | ? | - | - | + | + | ? |
| Wu Chao et al. (2018) | + | + | + | + | + | + | ? |
| Zhou Y-zhong et al. (2018) | + | + | + | + | + | + | ? |

The dark green cells (+) indicate a low risk of bias. The red cells (-) indicate a high risk of bias. The yellow cells (?) indicate an uncertain risk of bias. n/a, not applicable.
