# Supplementary material for: Adverse Events Associated With Treatment of Tripterygium wilfordii Hook F: A Quantitative Evidence Synthesis
Source: Front Pharmacol. 2019 Nov 6;10:1250. doi: 10.3389/fphar.2019.01250 (PMC6851843; doi:10.3389/fphar.2019.01250)
Supplement: Supplementary file 1 [file DataSheet_1.zip › Supplementary Table 2.DOCX]

**Supplementary Table 2.** Newcastle-Ottawa Scale (NOS) for quality assessment of non-RCT.

|  | **Selection** | **Comparability** | **Exposure/outcome** | **Overall star rating** |
| --- | --- | --- | --- | --- |
| Ao J-hua et al. (1994） | +++ | ++ | ++ | 7 |
| Jiang X-you et al. (1994) | ++ |  | ++ | 4 |
| Tao J-zang et al. (1995) | + | ++ | +++ | 6 |
| Ji S-ming et al. (1998) | +++ | ++ | ++ | 7 |
| Zhou J-ling et al. (1999) | +++ | ++ | ++ | 7 |
| Li L-feng et al. (2000) | + |  | +++ | 4 |
| Gao Y-ping et al. (2004) | ++ | + | ++ | 5 |
| Wang Jian et al. (2004) | +++ | ++ | ++ | 7 |
| Zhou J-hua et al. (2004) | +++ | ++ | ++ | 7 |
| Zhou X-ping et al. (2004) | +++ | ++ | ++ | 7 |
| Zou Xi et al. (2006) | + |  | +++ | 4 |
| Ren J-nan et al. (2007) | +++ |  | + | 4 |
| Mao L-ming et al. (2008) | ++ | ++ | ++ | 6 |
| Li J-guo et al. (2009) | +++ | ++ | ++ | 7 |
| Ji Wei et al. (2010) | +++ |  | + | 4 |
| Zhang Wen et al. (2010) | +++ | ++ | ++ | 7 |
| Chen Fang et al. (2012) | ++ |  | +++ | 5 |
| Liu S-shan et al. (2015) | +++ | ++ | ++ | 7 |
| Li Chen et al. (2017) | ++ |  | + | 3 |
| Wang Zhen et al. (2017) | +++ | ++ | ++ | 7 |
| Shang S-lai et al. (2018) | +++ | ++ | ++ | 7 |

A star system was used for allow a semi quantitative assessment of study quality. A study was awarded a maximum of one star for each numbered item within the selection and exposure categories. A maximum of two stars were awarded for comparability. The NOS ranges from zero to nine stars. We considered high-quality studies as those that achieved seven or more stars, medium-quality studies those with four to six stars, and poor-quality studies those with fewer than four stars.
