# Supplementary figures and images for: Adverse Events Associated With Treatment of Tripterygium wilfordii Hook F: A Quantitative Evidence Synthesis
Source: Front Pharmacol. 2019 Nov 6;10:1250. doi: 10.3389/fphar.2019.01250 (PMC6851843; doi:10.3389/fphar.2019.01250)

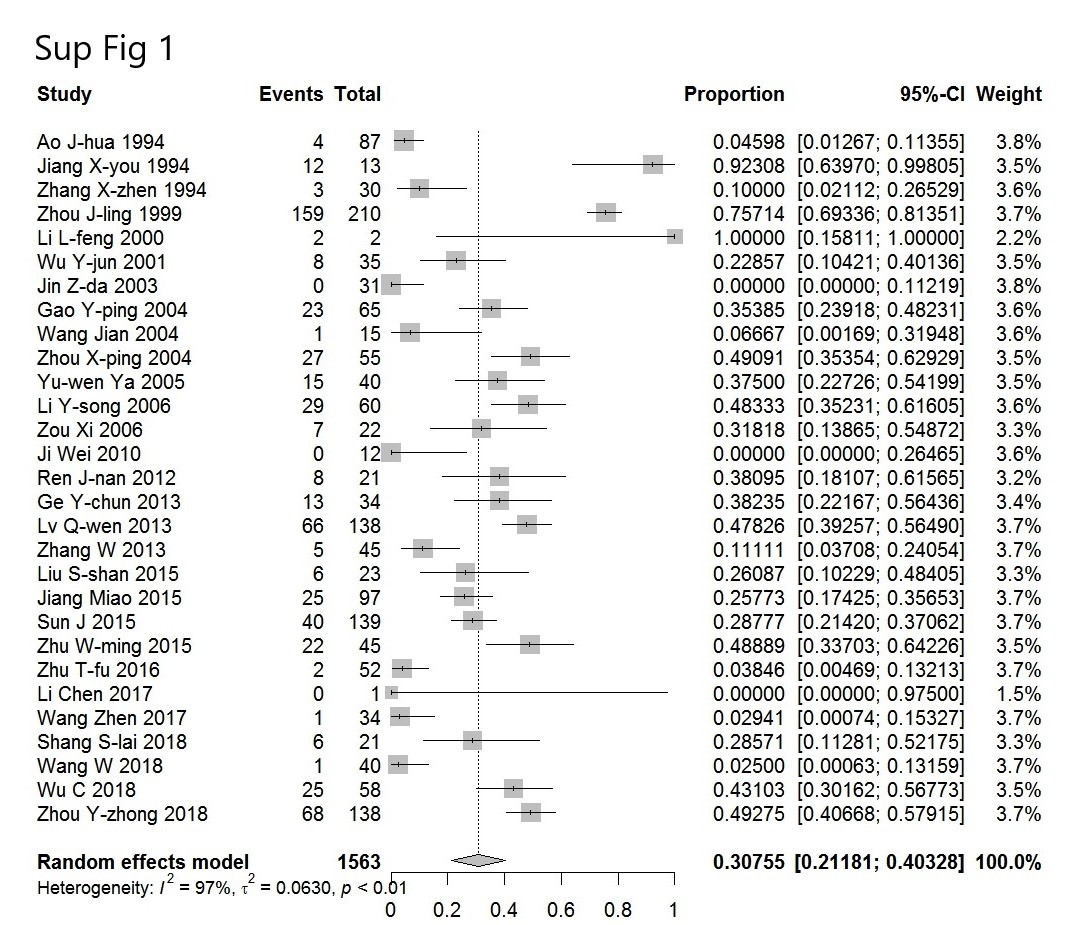

Supplement: Supplementary file 1 [file DataSheet_1.zip › Supplementary Figure 1.jpeg]

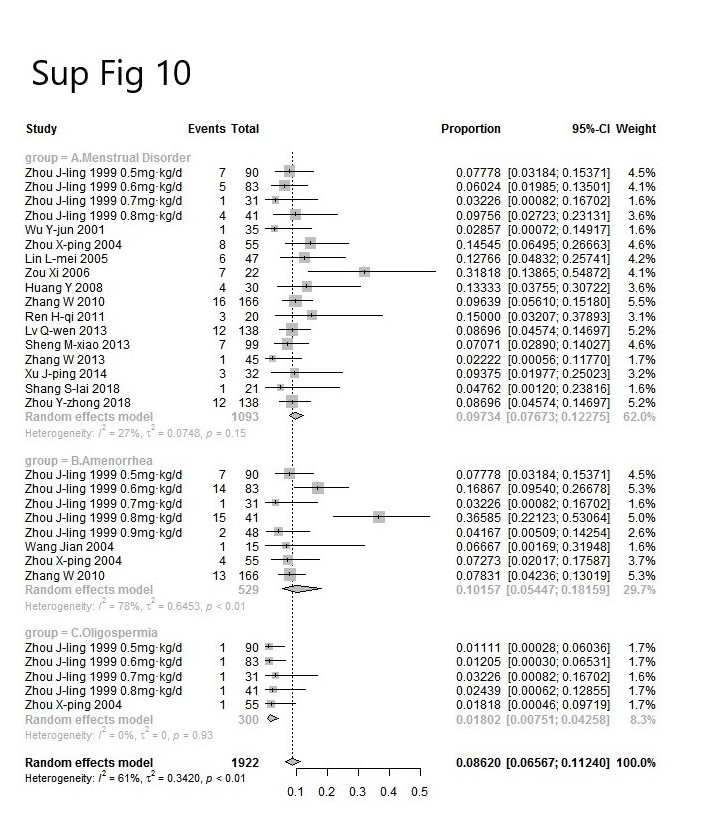

Supplement: Supplementary file 1 [file DataSheet_1.zip › Supplementary Figure 10.jpeg]

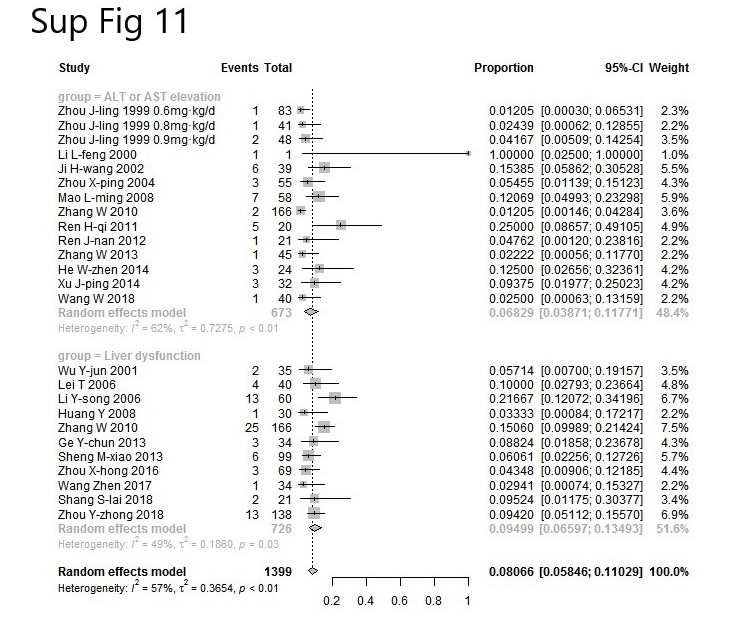

Supplement: Supplementary file 1 [file DataSheet_1.zip › Supplementary Figure 11.jpeg]

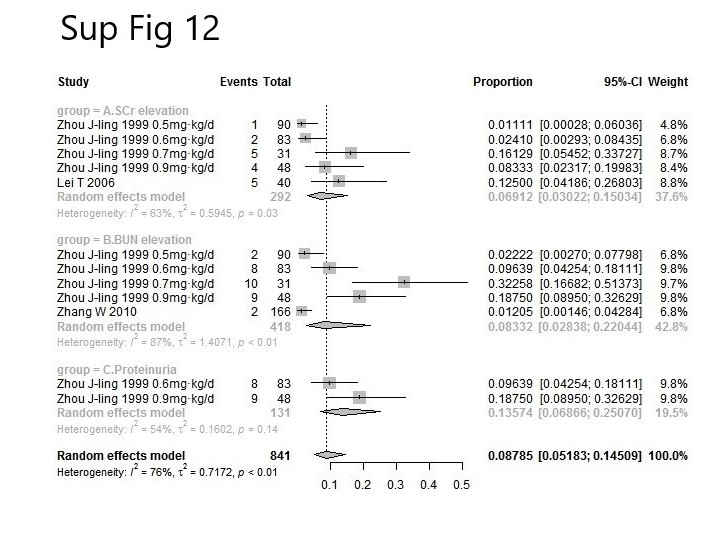

Supplement: Supplementary file 1 [file DataSheet_1.zip › Supplementary Figure 12.jpeg]

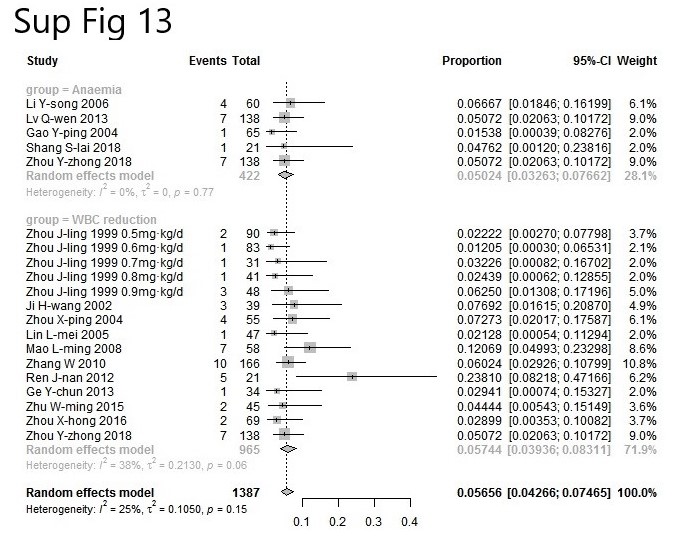

Supplement: Supplementary file 1 [file DataSheet_1.zip › Supplementary Figure 13.jpeg]

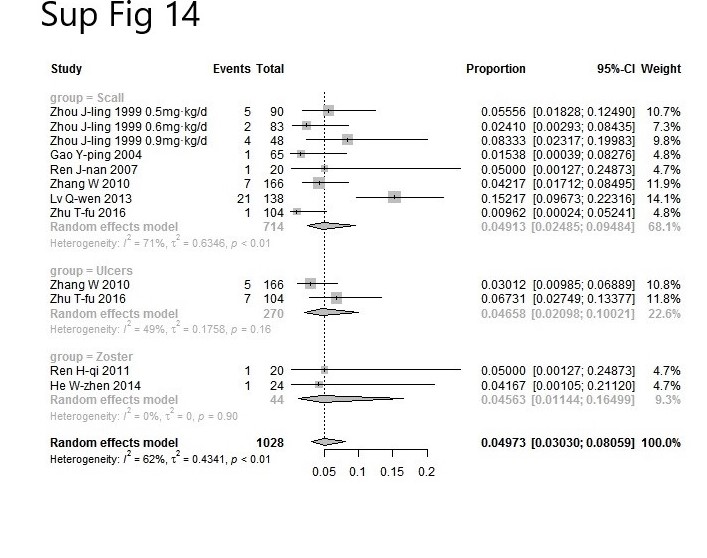

Supplement: Supplementary file 1 [file DataSheet_1.zip › Supplementary Figure 14.jpeg]

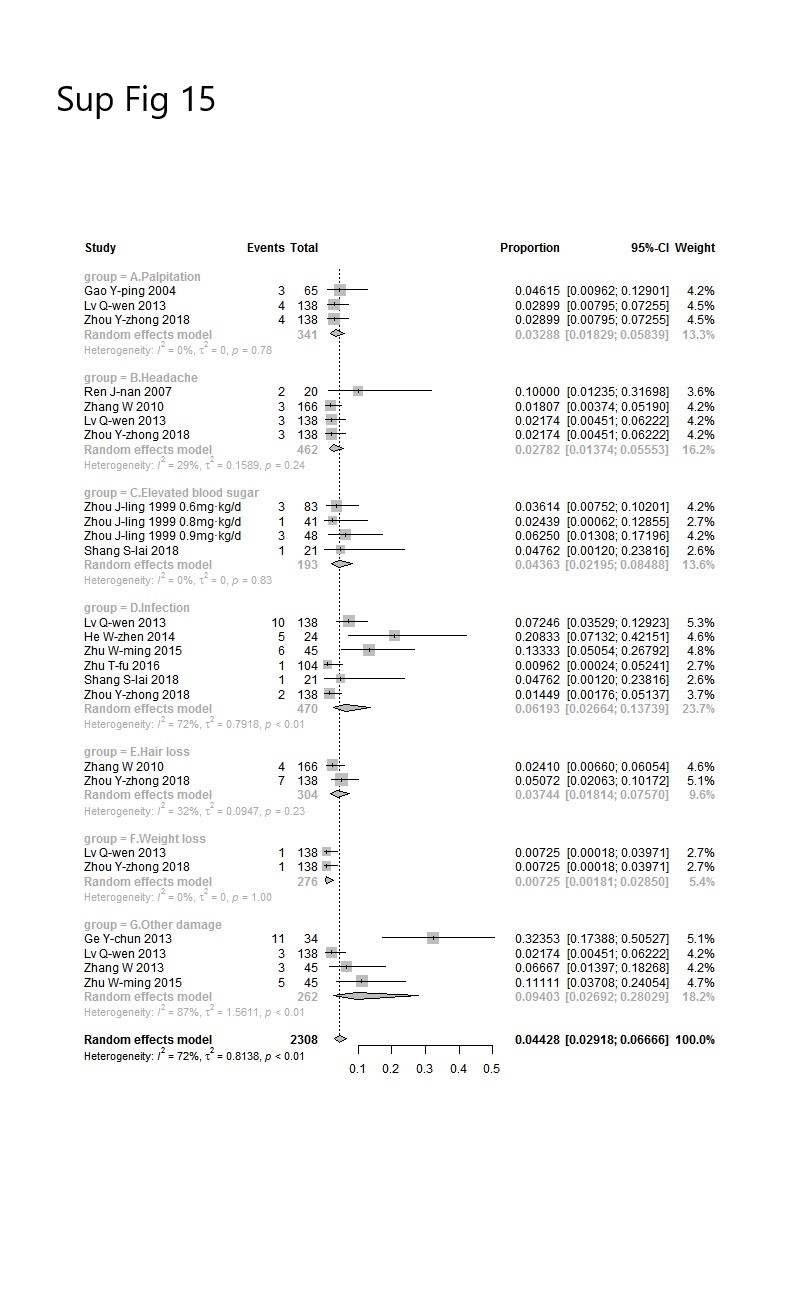

Supplement: Supplementary file 1 [file DataSheet_1.zip › Supplementary Figure 15.jpeg]

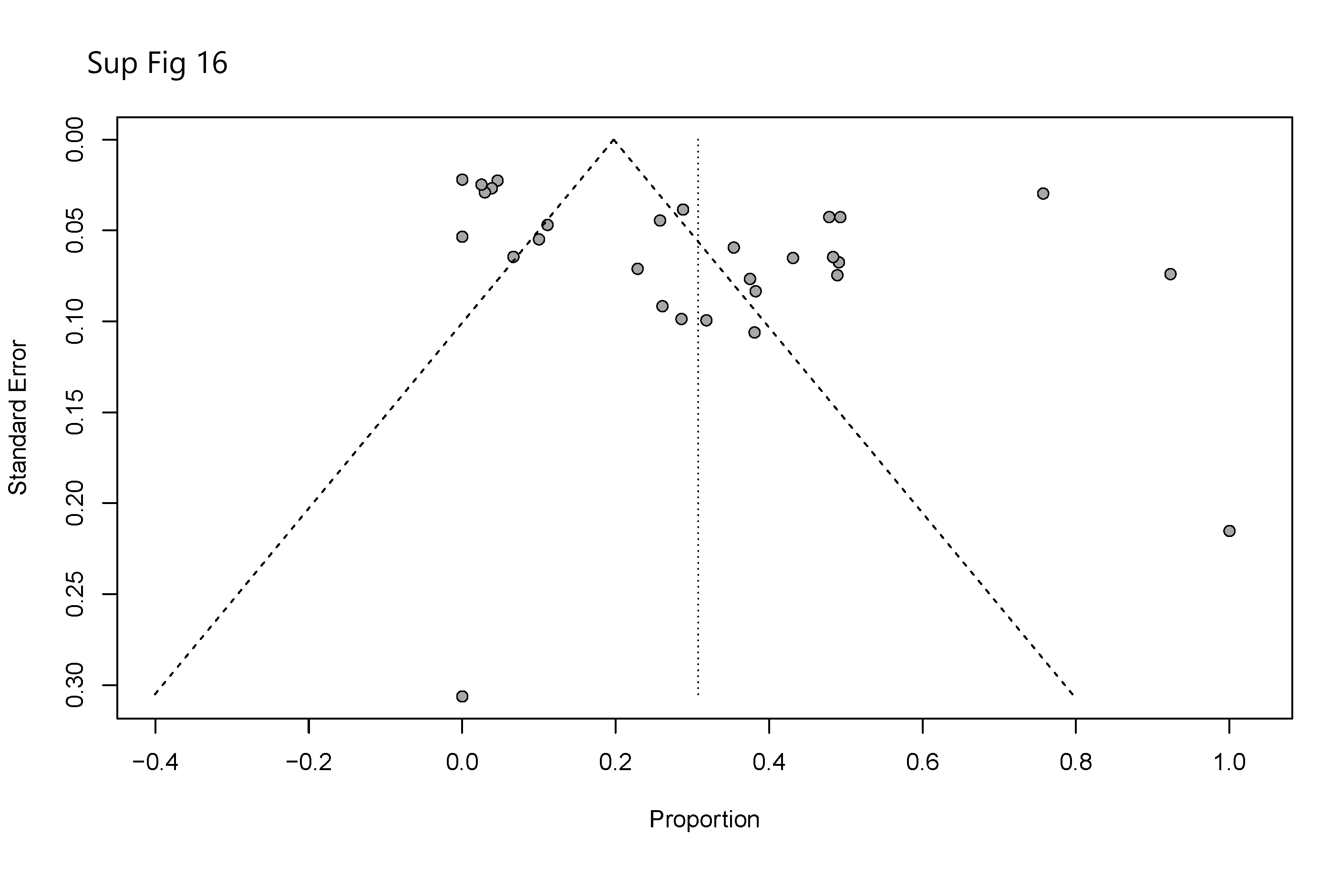

Supplement: Supplementary file 1 [file DataSheet_1.zip › Supplementary Figure 16.tiff]

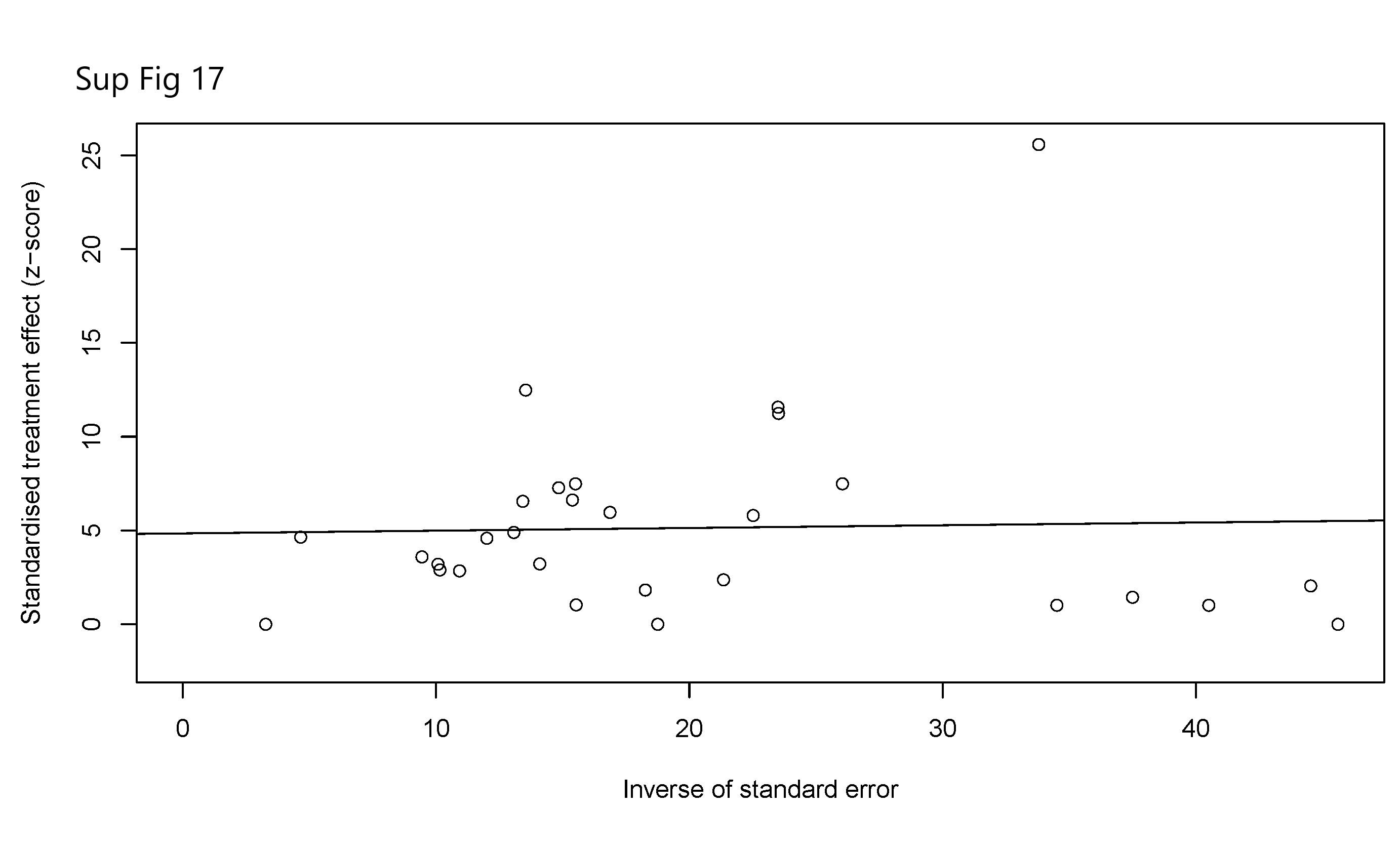

Supplement: Supplementary file 1 [file DataSheet_1.zip › Supplementary Figure 17.tiff]

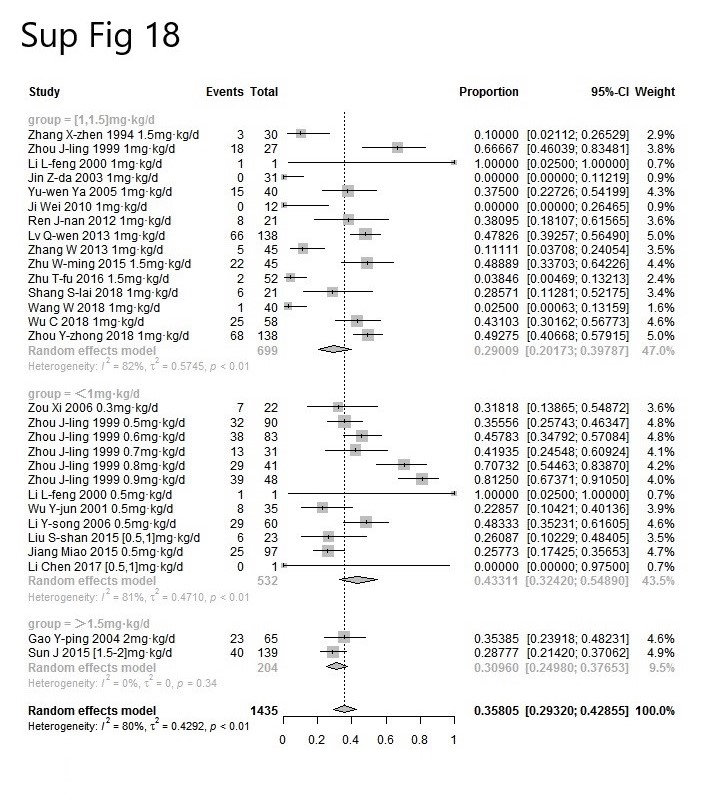

Supplement: Supplementary file 1 [file DataSheet_1.zip › Supplementary Figure 18.jpeg]

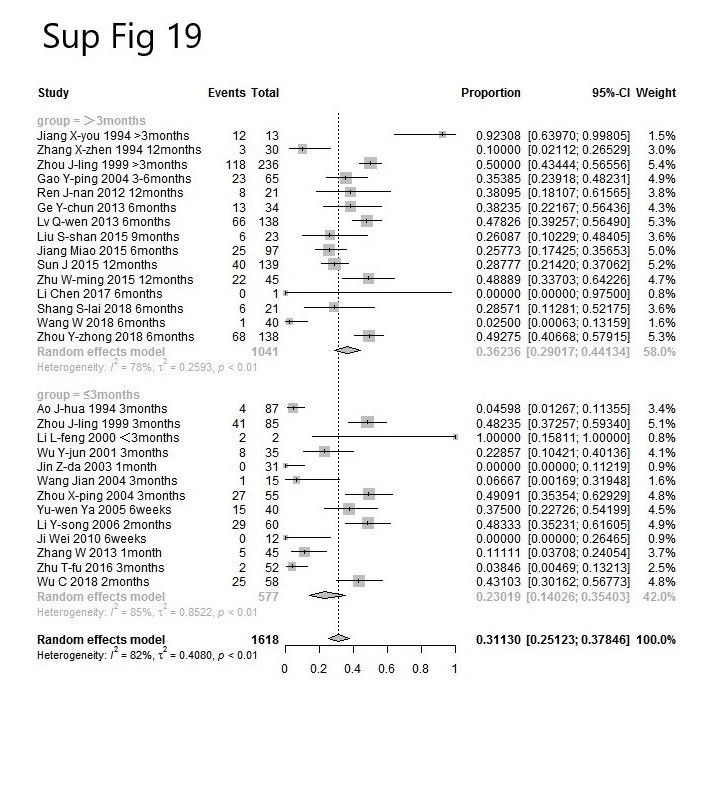

Supplement: Supplementary file 1 [file DataSheet_1.zip › Supplementary Figure 19.jpeg]

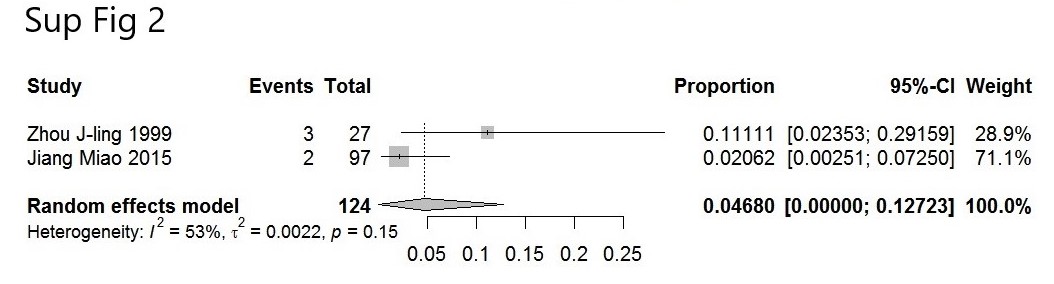

Supplement: Supplementary file 1 [file DataSheet_1.zip › Supplementary Figure 2.jpeg]

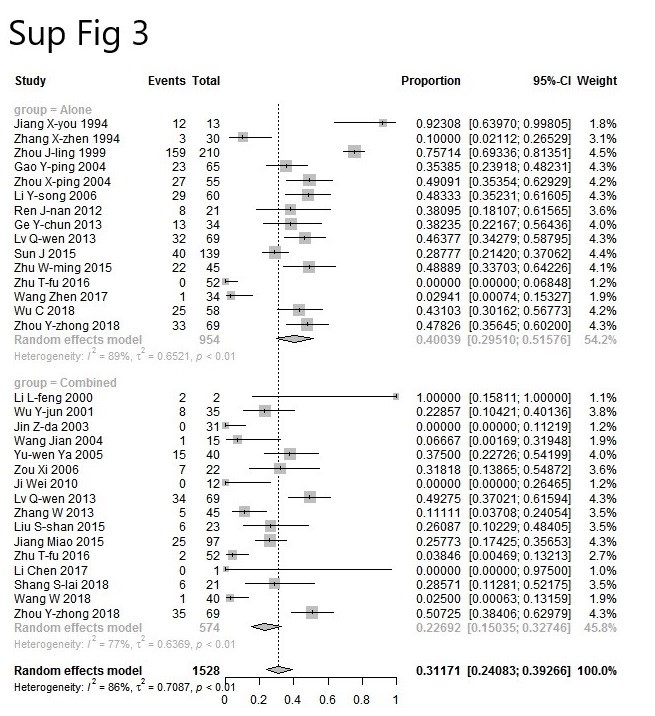

Supplement: Supplementary file 1 [file DataSheet_1.zip › Supplementary Figure 3.jpeg]

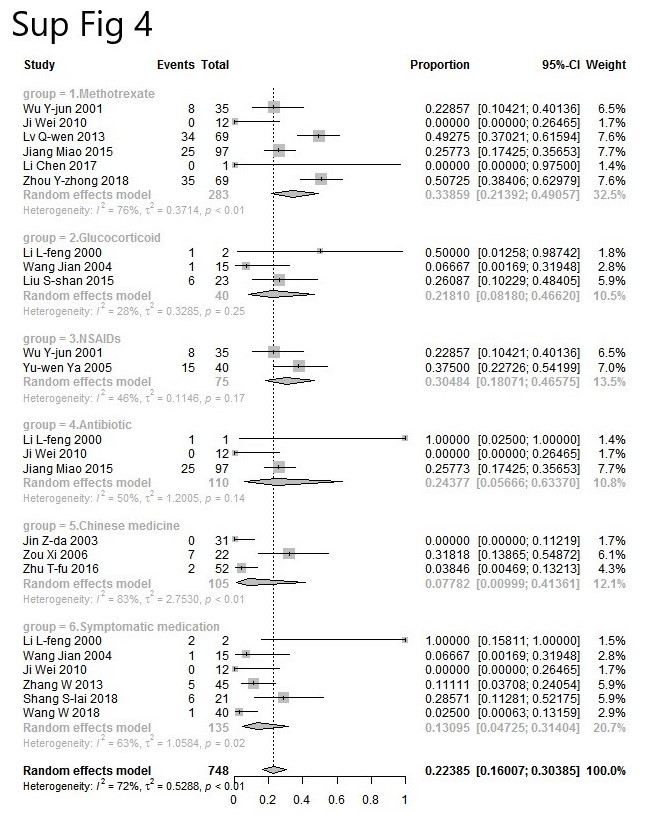

Supplement: Supplementary file 1 [file DataSheet_1.zip › Supplementary Figure 4.jpeg]

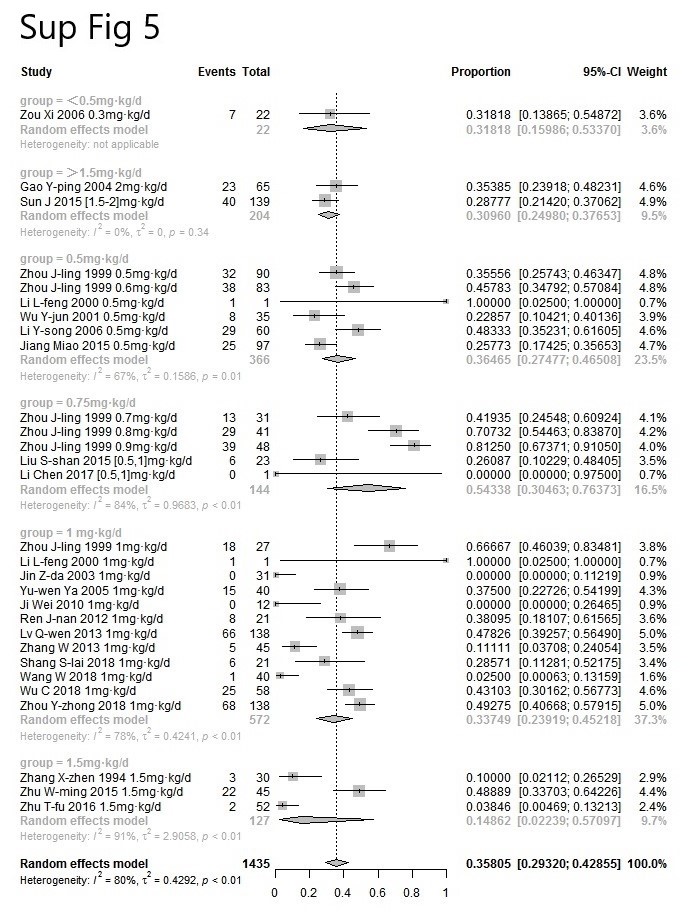

Supplement: Supplementary file 1 [file DataSheet_1.zip › Supplementary Figure 5.jpeg]

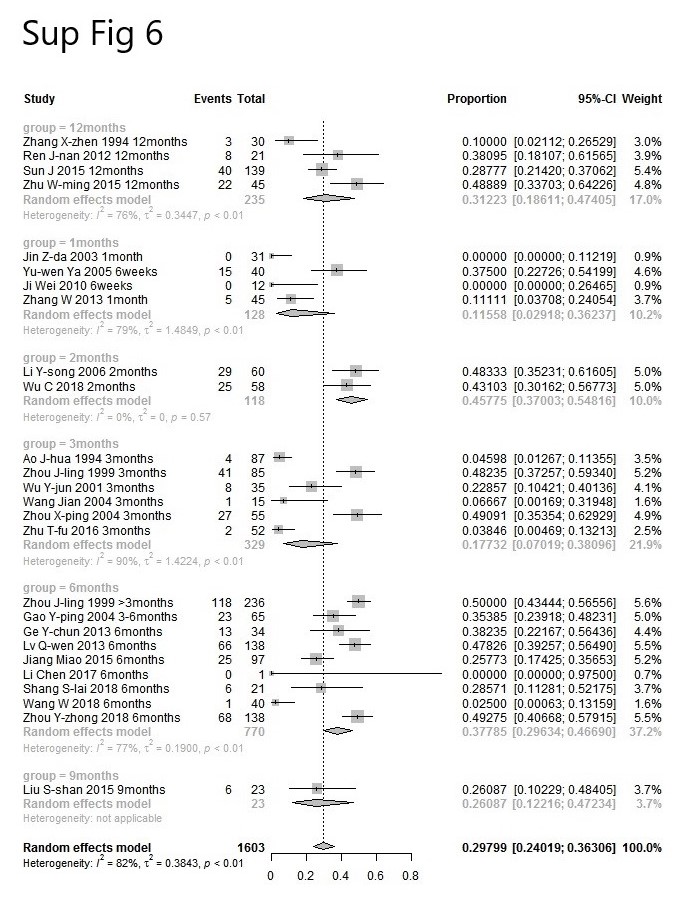

Supplement: Supplementary file 1 [file DataSheet_1.zip › Supplementary Figure 6.jpeg]

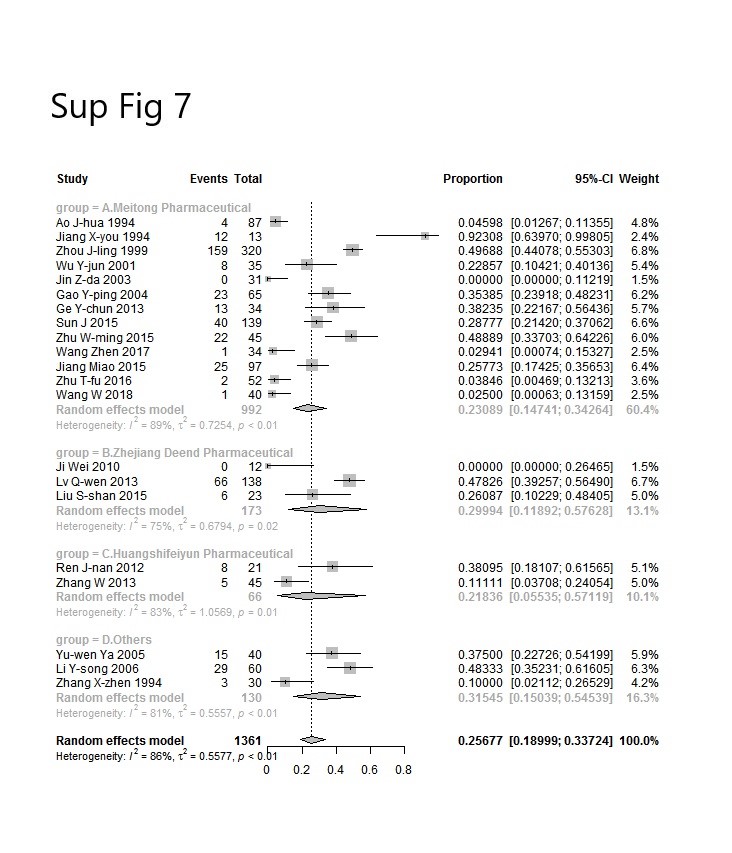

Supplement: Supplementary file 1 [file DataSheet_1.zip › Supplementary Figure 7.jpeg]

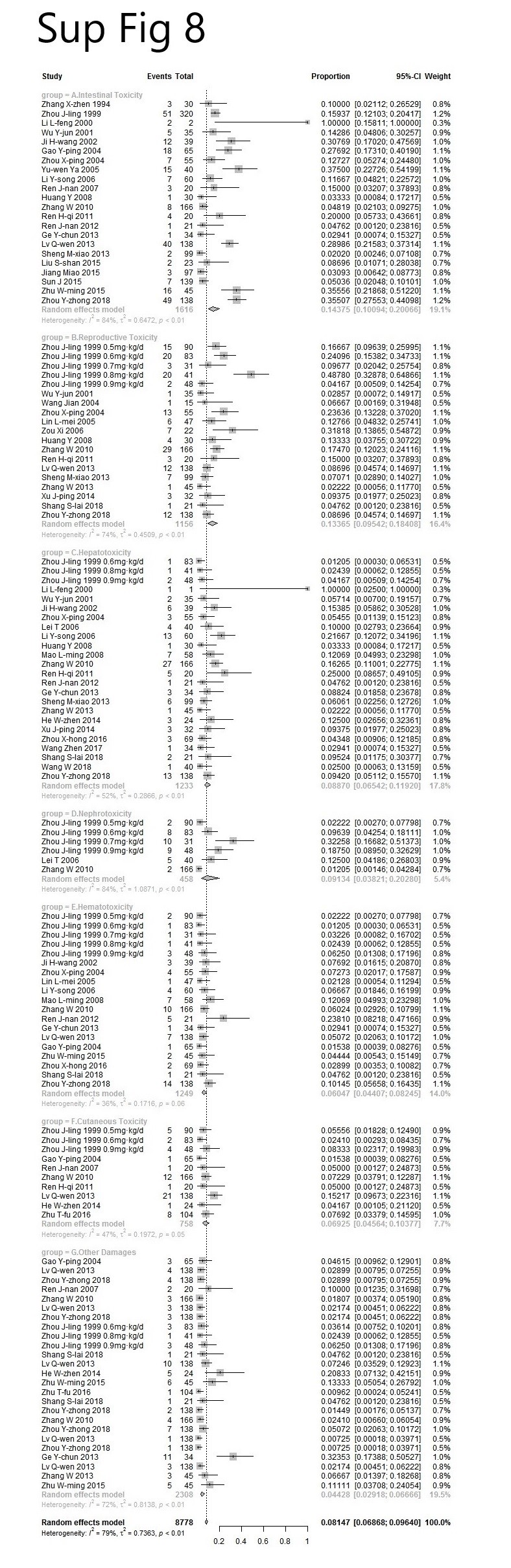

Supplement: Supplementary file 1 [file DataSheet_1.zip › Supplementary Figure 8.jpeg]

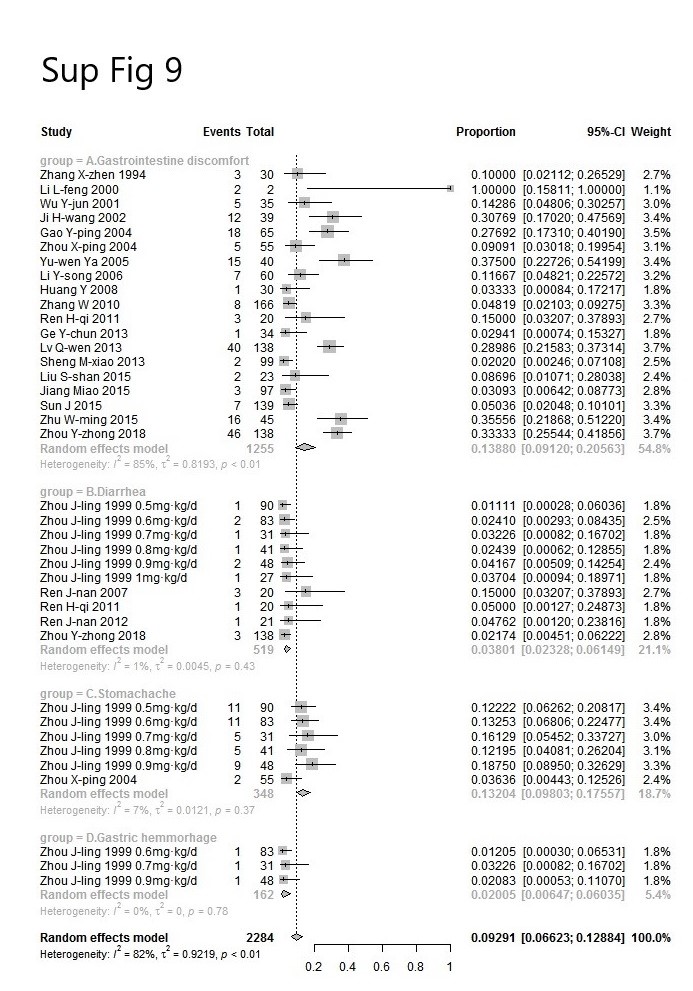

Supplement: Supplementary file 1 [file DataSheet_1.zip › Supplementary Figure 9.jpeg]
